# Natural Benzo/Acetophenones as Leads for New Synthetic Acetophenone Hybrids Containing a 1,2,3-Triazole Ring as Potential Antifouling Agents

**DOI:** 10.3390/md19120682

**Published:** 2021-11-29

**Authors:** Ana Rita Neves, Daniela Pereira, Catarina Gonçalves, Joana Cardoso, Eugénia Pinto, Vitor Vasconcelos, Madalena Pinto, Emília Sousa, Joana R. Almeida, Honorina Cidade, Marta Correia-da-Silva

**Affiliations:** 1 Laboratory of Organic and Pharmaceutical Chemistry, Department of Chemical Sciences, Faculty of Pharmacy, University of Porto, Rua Jorge Viterbo Ferreira, 228, 4050-313 Porto, Portugal; anarcneves92@gmail.com (A.R.N.); dmpereira@ff.up.pt (D.P.); up201603353@edu.ff.up.pt (J.C.); madalena@ff.up.pt (M.P.); esousa@ff.up.pt (E.S.); m_correiadasilva@ff.up.pt (M.C.-d.-S.); 2 CIIMAR—Interdisciplinary Centre of Marine and Environmental Research, University of Porto, Avenida General Norton de Matos, 4450-208 Matosinhos, Portugal; catarina.goncalves@ciimar.up.pt (C.G.); epinto@ff.up.pt (E.P.); vmvascon@fc.up.pt (V.V.); 3 Laboratory of Microbiology, Department of Biological Sciences, Faculty of Pharmacy, University of Porto, Rua de Jorge Viterbo Ferreira 228, 4050-313 Porto, Portugal; 4 Department of Biology, Faculty of Sciences, University of Porto, Rua do Campo Alegre, 4069-007 Porto, Portugal

**Keywords:** acetophenones, 1,2,3-triazole, click chemistry, antifouling, ecotoxicity, eco-friendly

## Abstract

Marine biofouling is a natural process that represents major economic, environmental, and health concerns. Some booster biocides have been used in biofouling control, however, they were found to accumulate in environmental compartments, showing negative effects on marine organisms. Therefore, it is urgent to develop new eco-friendly alternatives. Phenyl ketones, such as benzophenones and acetophenones, have been described as modulators of several biological activities, including antifouling activity (AF). In this work, acetophenones were combined with other chemical substrates through a 1,2,3-triazole ring, a strategy commonly used in Medicinal Chemistry. In our approach, a library of 14 new acetophenone–triazole hybrids was obtained through the copper(I)-catalyzed alkyne-azide cycloaddition “click” reaction. All of the synthesized compounds were evaluated against the settlement of a representative macrofouling species, *Mytilus galloprovincialis*, as well as on biofilm-forming marine microorganisms, including bacteria and fungi. The growth of the microalgae *Navicula* sp. was also evaluated after exposure to the most promising compounds. While compounds **6a, 7a**, and **9a** caused significant inhibition of the settlement of mussel larvae, compounds **3b**, **4b**, and **7b** were able to inhibit *Roseobacter litoralis* bacterial biofilm growth. Interestingly, acetophenone **7a** displayed activity against both mussel larvae and the microalgae *Navicula* sp., suggesting a complementary action of this compound against macro- and microfouling species. The most potent compounds (**6a**, **7a**, and **9a**) also showed to be less toxic to the non-target species *Artemia salina* than the biocide Econea^®^. Regarding both AF potency and ecotoxicity activity evaluation, acetophenones **7a** and **9a** were put forward in this work as promising eco-friendly AF agents.

## 1. Introduction

Biofouling is an inevitable natural phenomenon that occurs continually on marine vessels and other submerged structures. Propulsive fuel increase, required to overcome the increased drag created by fouled hulls, constitutes significant costs for the maritime industry, and there is a substantial industrial and commercial interest in controlling the biofouling process, namely by dry-docking, scraping, and re-painting hulls [1].

The natural process of marine biofouling starts with the deposition of organic material that encourages the attachment of marine bacteria, diatoms, and fungi, and the colonization of macroorganisms, such as macroalgae, sponges, cnidarians, polychaetes, mollusks, barnacles, bryozoans, and tunicates [2]. Therefore, it is important to target diverse levels of fouling when trying to efficiently combat biofouling.

Antifouling (AF) coatings, used to prevent biofouling, are of great environmental concern. Biocides, after being released from the AF coating, can accumulate in the environment, predominantly in areas where there is intense boating activity. Most biocides are persistent and do not undergo degradation rapidly, bioaccumulating through the food chain and showing negative effects in marine organisms. The deposition in sediments is also a concern, as they will be continually released into the water. Therefore, there is a renewed effort to develop harmless alternatives. In addition to the search of new eco-friendly AF agents, other technologies to prevent marine biofouling have been developed, such as silicone polymer AF coatings, biomimetic AF coatings, or photocatalytic AF coatings; however, these technologies are hard to apply. Therefore, the development of AF compounds with low environmental impact remains one of the most useful strategies in the development of new effective AF coatings [3].

**Figure 1:**
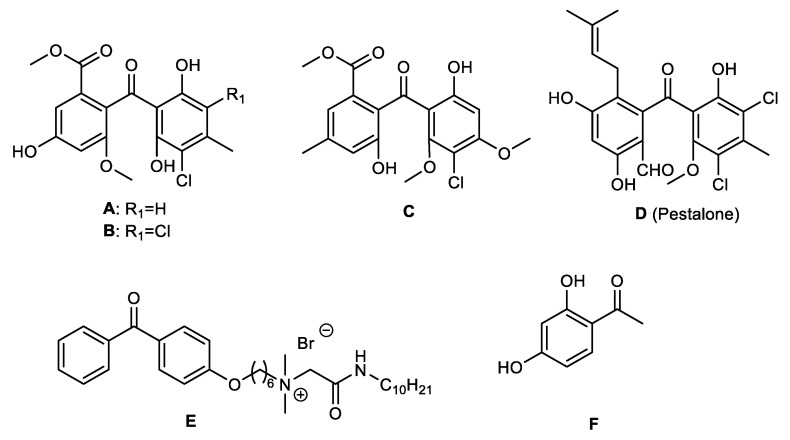
Some benzo/acetophenones with promising antimicrobial and AF activities.

In recent years, marine phenyl ketones, such as benzophenones **A**–**D**, have been reported in the literature as antibacterial and antifungal agents (Figure 1) [4,5,6]. Moreover, the benzophenone scaffold has been used for the preparation of some AF coatings (**E**, Figure 1) [7]. Molecular simplification of the benzophenone structure has also been applied in the development of promising AF agents: 2,4-dihydroxyacetophenone (**F**, Figure 1) was shown to significantly inhibit the spore attachment of a green fouling alga (*Ulva pertusa*) and, after being incorporated in a controlled depletion paint, a significant decrease in fouling biomass was observed [8]. Therefore, phenyl ketones, such as benzophenones and acetophenones, show potential as new leads to develop eco-friendly and sustainable AF agents for the marine industry.

In this work, several acetophenones were synthesized, and the AF activity was assessed against the settlement of *Mytilus galloprovincialis*, a heavy macrofouler, five strains of biofilm-forming marine bacteria (*Cobetia marina*, *Vibrio harveyi*, *Pseudoalteromonas atlantica*, *Halomonas aquamarina*, and *Roseobacter litoralis*), a marine diatom (*Navicula* sp.), and three fungal strains (*Candida albicans*, *Aspergillus fumigatus*, and *Trichophyton rubrum*). The ecotoxicity and bioaccumulative potential of the most promising compounds was also evaluated in the discovery of eco-friendly compounds.

## 2. Results and Discussion

### 2.1. Synthesis and Structure Elucidation

Combining different bioactive ligands/pharmacophores into a single molecule is a strategy currently employed in Medicinal Chemistry even for AF activity [9,10]. Triazole derivatives have undeniable importance in Medicinal Chemistry, displaying several bioactivities, such as antimicrobial, which includes the family of the so-called “azoles” used in the treatment of fungal infections [11]. The incorporation of a 1,2,3-triazole ring in the benzophenone and acetophenone scaffolds was used to generate compounds with antimicrobial activity [12,13]. Moreover, the presence of triazoles was already associated to AF activity of biocides [14]. For instance, bromotyramine hybrids containing a 1,2,3-triazole ring were described as inhibitors of biofilm formed by marine bacteria [15]. Recently, previous research developed by our group has identified a chalcone glycoside with a 1,2,3-triazole moiety with promising AF activity against macro and microfouling species, without ecotoxicity to a non-target marine organism [16].

**Figure 2:**
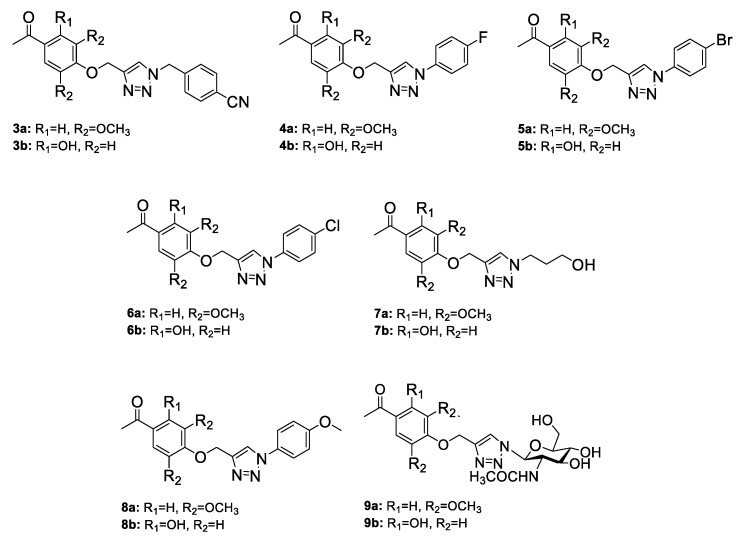
Structures of compounds **3a**–**9b**.

Based on these data, and considering the importance of both 1,2,3-triazole and benzo/acetophenone moieties, a series of 14 new 1,2,3-triazole tethered acetophenones (**3a**–**9b**) was synthesized (Figure 2).

**Scheme 1:**
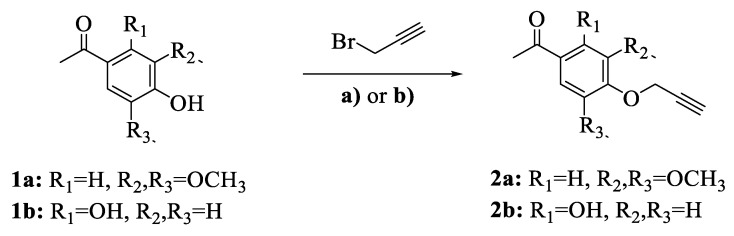
Synthesis of propargyl acetophenones **2a** and **2b**. **a**) CsCO_3_, acetone, reflux, 3 h, 84% yield; **b**) K_2_CO_3_, acetone, reflux, 3 h, 76% yield.

The 1,2,3-triazoles are easily obtained through the copper-catalyzed alkyne-azide Huisgen cycloaddition (CuAAC) reaction, a well-known example of a class of chemical reactions called “click reactions” [17,18]. The first step to obtaining triazole-linked acetophenone derivatives **3a**–**9b** was the propargylation of acetophenones **1a** and **1b** with propargyl bromide, in the presence of either anhydrous CsCO_3_ or K_2_CO_3_, respectively, which gave compounds **2a** and **2b** in 84% and 76% yield, respectively (Scheme 1).

The incorporation of the triazole was accomplished by the CuAAC reaction (Table 1). To obtain structure–activity relationship (SAR) information, several structure-related acetophenones with diversified substitution patterns were explored: aromatic nitriles (**3a** and **3b**), since isocyanides, their isomers, were extensively studied in AF activity [19]; aromatic halogens to combine with acetophenones (**4a**–**6b**) and obtain 1,4 disubstituted 1,2,3-triazoles as the literature reports hybrids with these substitutions as having antimicrobial activity [20,21,22]; and the other two substituents, namely an aliphatic alcohol (**7a** and **7b**) and a glucose acetamide (**9a** and **9b**) were also included for SAR studies.

Acetophenones **2a** and **2b** reacted with the selected azides in the presence of catalytically copper (I) ions formed by the reduction of copper (II) from CuSO_4_ by sodium ascorbate, to afford derivatives **3a**–**9b** in 30–84% yields (Table 1).

The newly synthesized compounds **3a**–**9b** were characterized by infrared spectroscopy (IR), nuclear magnetic resonance (NMR), and high-resolution mass spectrometry (HRMS) (Supplementary material, Figures S1–S28). Compounds **2a** and **2b** were already described in the literature and NMR data are in accordance with the data already published [23,24].

The NMR spectra of compounds **3a**–**9b** showed the characteristic signals of the acetophenone scaffold. The signals corresponding to the triazole ring were also observed (δ_H_-triazole: 7.71–9.00 s, δ_C_-H triazole: 134.7–145.8 and 118.2–121.2). Regarding compounds **3a**–**6b**, **8a**, and **8b**, signals corresponding to the aromatic protons were observed. Additionally, a carbon signal corresponding to C≡N (δ_C_ 118.2 and 118.6, respectively) was observed for compounds **3a** and **3b**. The NMR spectra of compounds **9a** and **9b** showed several proton and carbon signals corresponding to the acetamide glucose moiety. Aliphatic protons and carbons were observed in the NMR spectra of compounds **7a** and **7b**, and proton and carbons signals assigned to the methoxy group of compounds **8a** and **8b** were observed in the NMR spectra of these compounds.

### 2.2. Mussel Larvae Anti-Settlement Activity

**Figure 3:**
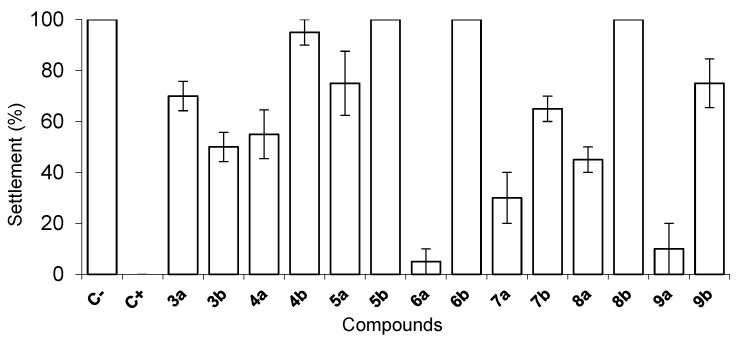
Anti-settlement activity of compounds **3a**–**9b** at 50 μM towards plantigrade larvae of the mussel *Mytilus galloprovincialis*. C-: DMSO control (0.1%); C+: 5 μM CuSO_4_ as positive control.

The presence of macrofouling species has a dominant influence on ship drag [25], increasing fuel consumption. The larvae of some species of bryozoans, polychaetes, mollusks, and some other biofoulers may adhere even before biofilm formation. Mussel plantigrade larvae are highly specialized in adhesion to the submerged surfaces and the fixation is made through the production of byssal threads. Therefore, mussel plantigrade larvae were selected to assess the AF activity of the newly synthesized 1,2,3-triazolylacetophenones (**3a**–**9b**). The ability of the synthesized compounds to inhibit the settlement of *Mytilus galloprovincialis* larvae was first screened at 50 μM (Figure 3).

Among the 14 tested compounds, **6a**, **7a**, and **9a** presented a percentage of settlement ≤ 40% and were selected as positive hits for dose–response studies to determine the EC_50_ and LC_50_/EC_50_ values regarding anti-settlement activity (Table 2). In general, when comparing the results of compounds with the same substitution on the 1,2,3-triazole ring, it was found that the presence of two methoxy groups at C-3′ and C-5′ on the phenyl ketone core is more beneficial for the mussel larvae anti-settlement activity than the presence of hydroxyl groups at C-2′.

The three compounds presented an EC_50_ < 25 µg·mL^−1^, a value recommended by the U.S. Navy program for anti-foulants [26]. Acetophenone derivative **9a** (EC_50_ = 9.94 µg·mL^−1^), containing an acetamide glucose moiety was the most effective larval settlement inhibitor, followed by compounds **6a** (EC_50_ = 11.20 µg·mL^−1^) with an aromatic chlorine, and **7a** (EC_50_ = 13.46 µg·mL^−1^) with an aliphatic alcohol. Considering toxicity, none of these compounds caused mortality to the target species *M. galloprovincialis* plantigrades to the highest concentration tested (200 μM).

### 2.3. Biofilm-Forming Marine Microorganism Growth Inhibitory Activity

**Figure 4:**
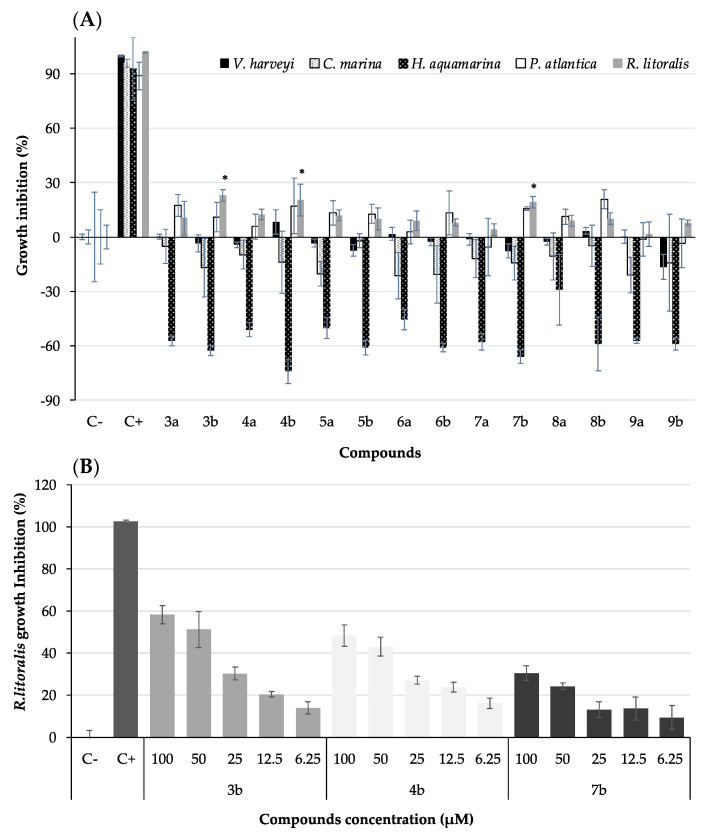
Bacterial growth inhibition screening at 15 µM (**A**) and dose–response (**B**) of acetophenone derivatives towards five biofilm-forming marine bacteria: *Vibrio harveyi*, *Cobetia marina*, *Halomonas aquamarina*, *Pseudoalteromonas atlantica*, and *Roseobacter litoralis*. C-: negative control with a solution of marine broth with 0.1% DMSO; C+: positive control with penicillin–streptomycin–neomycin stabilized solution. * indicates significant differences against the C- (Dunnett test, *p* < 0.01).

The settlement of some macroorganisms might be enhanced by the presence of microbial biofilms present in submerged surfaces [27]. Microbial biofilms composed of bacteria, fungi, diatoms, unicellular algae, and protozoa, represent an important component of fouling communities. Therefore, synthesized acetophenones were also evaluated for their ability to inhibit the growth of marine biofilm-forming bacteria (*Vibrio harveyi*, *Cobetia marina*, *Halomonas aquamarina*, *Pseudoalteromonas atlantica,* and *Roseobacter litoralis*), fungi (*Candida albicans*, *Aspergillus fumigatus*, and *Trichophyton rubrum),* and microalgae (*Navicula* sp.). Results showed that compounds **3b**, **4b**, and **7b** were able to significantly inhibit *R. litoralis* biofilm growth (Figure 4A). In contrast to the mussel larvae anti-settlement activity, the presence of hydroxyl groups at C-2′ on the phenyl ketone core seems to be more beneficial for the antibacterial activity.

The dose–response activities of **3b** and **4b** were the most relevant, with an EC_50_ of 49.81 µM; 17.35 µg·mL^−1^ (CI: 39.99–65.63) and 105.02 µM; 34.38 µg·mL^−1^ (CI: 67.05–235.78), respectively (Figure 4B). EC_50_ was not possible to estimate for compound **7b**, given the low activity at the higher concentrations tested.

Fungi are also important players in the marine biofouling colonization cascade, as they are also able to produce biofilms. Therefore, to evaluate the AF potential of compounds **3a**–**9b** against microfouling species, their antifungal activity was evaluated against three biofilm-forming fungi species, *Candida albicans* [28], *Aspergillus fumigatus* [29], and *Trichophyton rubrum* [30], by determining minimum inhibitory concentration (MIC). None of the compounds tested showed activity against the fungal strains, with MICs higher than the maximum tested concentration (128 µg·mL^−1^).

**Figure 5:**
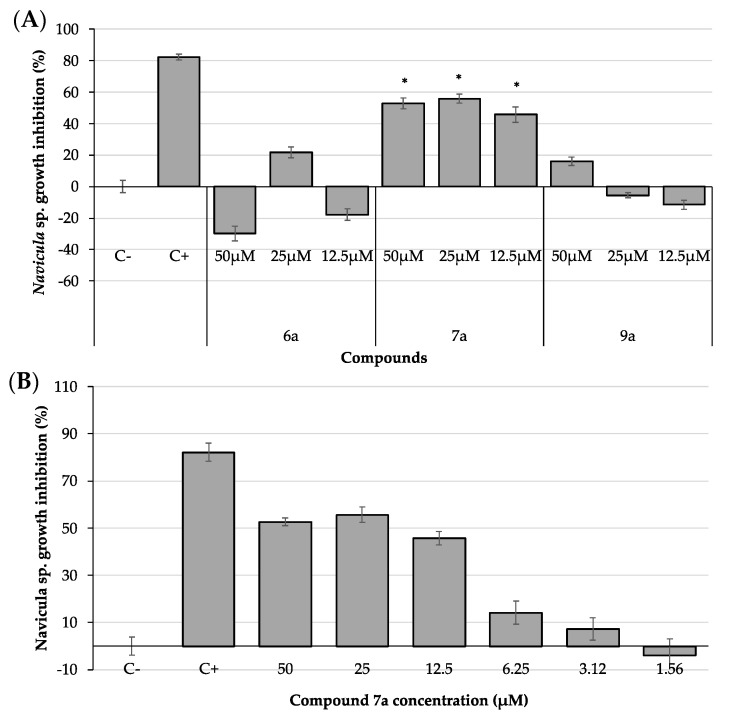
*Navicula* sp. growth inhibition screening of the most promising compounds **6a**, **7a**, and **9a** at 15 µM (**A**) and dose–response concentration of compound **7a** (**B**). C-: negative control with f/2 medium with 0.1% dimethyl sulfoxide (DMSO); C+: positive control with cycloheximide solution (3.55 µM). * Indicates significant differences against the C- (Dunnett test, *p* < 0.01).

Marine diatoms colonize very quickly and effectively submerged surfaces by secreting adhesive extracellular polymer substances, and thus are a good representative of fouling microalgae. The ability to inhibit the growth of the biofilm-forming marine diatom *Navicula* sp. was also evaluated but only for compounds with the most significant AF activity (**6a**, **7a**, and **9a**). From the three tested compounds, acetophenone **7a** showed inhibitory activity against *Navicula* sp. growth (Figure 5), with an EC_50_ of 26.73 µM; 8.96 µg·mL^−1^ (CI: 14.25–111.86). The activity obtained for this compound against *Navicula* sp., as well as the anti-settlement of the macrofouling species *M. galloprovincialis,* suggests a complementary action of **7a** at different levels of the biofouling process, reinforcing its potential as an AF agent.

### 2.4. Artemia Salina Ecotoxicity Bioassay

**Figure 6:**
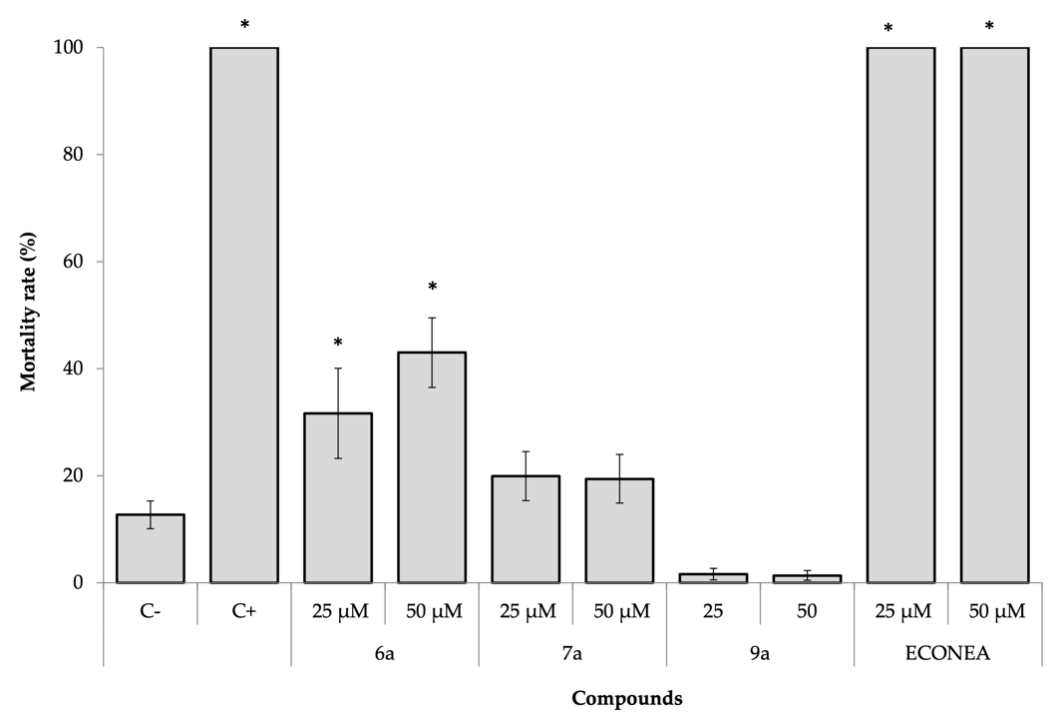
Mortality rate of *Artemia salina* nauplii after 48 h of exposure to compounds **6a**, **7a**, and **9a**. C-: negative control with 0.1% DMSO in filtered seawater. C+: positive control K_2_Cr_2_O_7_ at 13.6 μM. ECONEA^®^ was added for comparative purposes [33]. * Indicates significant differences against C- (Dunnett test, *p* < 0.01).

The most active compounds (**6a**, **7a**, and **9a**,) were further submitted to ecotoxicity assays against non-target organisms. The standard ecotoxicity bioassay using the brine shrimp *Artemia salina* was used due to its easy culture, short generation time, cosmopolitan distribution, and commercial availability of their eggs in the latent form [31,32]. All tested compounds were found to be less toxic to *Artemia salina* at both concentrations tested (25 and 50 μM) than the commercial biocide ECONEA^®^ (100% lethality) [33], being acetophenones **7a** and **9a** mortality rates not significantly different from negative control (Figure 6).

### 2.5. In Silico Evaluation of Bioaccumulation Potential

One of the major concerns on new AF agents is the potential bioaccumulation in marine organisms. Compounds are considered potential bioaccumulative if the LogKow (octanol-water partition coefficient) is higher or equal to 3. Therefore, in silico prediction of LogKow values was calculated for the most promising compounds **7a** and **9a**. Both compounds presented a LogKow value lower than 3 (0.38 for **7a** and −2.32 for **9a**), suggesting their low potential for bioaccumulation [34].

## 3. Materials and Methods

### 3.1. General Methods

Reactions were monitored by analytical thin-layer chromatography (TLC). Purifications of compounds were carried out by flash column chromatography using Macherey-Nagel silica gel 60 (0.04–0.063 mm) and crystallization. Melting points were obtained in a Köfler microscope and are uncorrected. ^1^H and ^13^C NMR spectra were taken in CDCl_3_ or DMSO-d_6_ at room temperature, on Bruker Avance 300 instruments (300.13 MHz for ^1^H and 75.47 MHz for ^13^C). Chemical shifts are expressed in δ (ppm) values relative to tetramethylsilane (TMS) as an internal reference; ^13^C NMR assignments were made by 2D (HSQC and HMBC) NMR experiments (long-range ^13^C–^1^H coupling constants were optimized to 7 Hz). HRMS mass spectra were performed on an ApexQe FT-ICR MS (Bruker Daltonics, Billerica, MA, USA), equipped with a 7T actively shielded magnet, at C.A.C.T.I. University of Vigo, Spain. Ions were generated using a Combi MALDI-electrospray ionization (ESI) source. Acetosyringone was purchased from TCI. Moreover, 2,4-dihydroxyacetophenone, 80 wt% propargyl bromide in toluene, ~0.5 M solution of 4-(azidomethyl)benzonitrile in *tert*-butyl methyl ether, ~0.5 M solution of 1-azido-4-fluorobenzene in *tert*-butyl methyl ether, ~0.5 M solution of 1-azido-4-bromo benzene in *tert*-butyl methyl ether, ~0.5 M solution of 1-azido-4-chloro benzene in *tert*-butyl methyl ether, 3-azido-1-propanol, and ~0.5 M solution of 4-azidoanisole in *tert*-butyl methyl ether were purchased from Sigma-Aldrich. The 2-azidoethyl 2-acetamido-2-deoxy-*β*-d-glucopyranosyl azide was purchased from Synthose.

### 3.2. Synthesis and Structure Elucidation

#### 3.2.1. Synthesis of Propargyloxy Acetophenone **2a** and **2b**

Compounds **2a** and **2b** were synthesized and characterized following previously reported methods and ^1^H and ^13^C NMR data were in accordance with the previously reported [24] and [16,23], respectively.

#### 3.2.2. Synthesis of Triazolyl Acetophenones 3**a**–9**b**

A solution of sodium ascorbate (0.13–1.2 g, 0.64–6.0 mmol) and CuSO_4_·5H_2_O (0.10–0.75 g, 0.64–3.0 mmol) in 5 mL of water was added to a solution of 1-(3,5-dimethoxy-4-(prop-2-yn-1-yloxy)phenyl)ethan-1-one (0.15–0.35 g, 0.64–1.45 mmol) or 1-(2-hydroxy-4-(prop-2-yn-1-yloxy)phenyl)ethan-1-one (0.10–0.30 g, 0.53–1.58 mmol) in 10 mL of THF. After 10 min under stirring, the respective azide was added, and the reaction mixture was left at room temperature for 15 min to 24 h. THF was evaporated and the residue was diluted with water and extracted twice with CH_2_Cl_2_. The organic phase was dried over anhydrous sodium sulfate, filtered, and evaporated until dry.

4-((4-((4-Acetyl-2,6-dimethoxyphenoxy)methyl)-1*H*-1,2,3-triazol-1-yl)methyl)benzonitrile (**3a**). Moreover, ~0.5 M solution of 4-(azidomethyl)benzonitrile in *tert*-butyl methyl ether (1.4 mL, 0.74 mmol). The solid obtained was further purified through crystallization with ethyl acetate (0.16 g, 0.41 mmol, 65% yield). M.p. (ethyl acetate): 149.5–149.8 °C; IR (KBr) υ_max_: 3440, 3124, 3009, 2958, 2842, 2230, 1676, 1586, 1504, 1457, 1414, 1331, 1127, 955, 780 cm^−1^; ^1^H NMR (CDCl_3_, 300.13 MHz) δ: 7.71 (1H, brs, H-3″), 7.66 (2H, d, *J* = 8.2 Hz, H-4‴, H-6‴), 7.32 (2H, d, *J* = 8.2 Hz, H-3‴, H-7‴), 7.18 (2H, s, H-2′, H-6′), 5.59 (2H, s, H-1‴), 5.26 (2H, s, H-1″), 3.85 (6H, s, 3′,5′-OCH_3_), 2.58 (3H, s, H-2) ppm; ^13^C NMR (CDCl_3_, 75.47 MHz) δ: 197.0 (C1), 153.3 (C3′, C5′), 140.7 (C2″), 139.9 (C4′), 133.1 (C4‴, C6‴), 133.0 (C1′), 128.5 (C3‴, C7‴), 118.2 (C3″, 5‴-CN), 112.9 (C5‴), 105.8 (C2′, C6′), 66.4 (C1″), 56.4 (3′,5′-OCH_3_), 53.6 (C1‴), 26.5 (C2) ppm; HRMS (ESI^+^) *m*/*z* calcd. for C_21_H_21_N_4_O_4_ [M + H^+^] 393.1557, found 393.1552.

4-((4-((4-Acetyl-3-hydroxyphenoxy)methyl)-1*H*-1,2,3-triazol-1-yl)methyl)benzonitrile (**3b**). ~0.5 M Solution of 4-(azidomethyl)benzonitrile in *tert*-butyl methyl ether (1.06 mL, 0.53 mmol). The obtained solid was purified through flash column chromatography (*n*-hexane:ethyl acetate, 7:3) (0.154 g, 0.44 mmol, 84% yield). M.p. (*n*-hexane:ethyl acetate): 153.8–155.6 °C; IR (KBr) υ_max_: 3410, 3124, 3084, 2935, 2880, 2232, 1632, 1575, 1506, 1368, 1251, 850, 829 cm^−1^; ^1^H NMR (DMSO-d_6_, 300.13 MHz) δ: 12.62 (1H, s, OH-2′), 8.37 (1H, s, H-3″), 7.86 (2H, d, *J* = 8.2 Hz, H-4‴, H-6‴), 7.84 (1H, d, *J* = 8.7 Hz, H-6′), 7.47 (2H, d, *J* = 8.2 Hz, H-3‴, H-7‴), 6.62–6.57 (2H, m, H-3′, H-5′), 5.75 (2H, s, H-1‴), 5.23 (2H, s, H-1″), 2.56 (3H, s, H-2) ppm; ^13^C NMR (DMSO-d_6_, 75.47 MHz) δ: 203.3 (C1), 164.4, 164.0 (C2′, C4′), 142.5 (C2″), 141.4 (C2‴), 133.4 (C6′), 132.8 (C4‴, C6‴), 128.8 (C3‴, C7‴), 125.4 (C3″), 118.6 (5‴-CN), 114.0 (C1′), 111.0 (C5‴), 107.7 (C5′), 101.7 (C3′), 61.5 (C1″), 52.3 (C1‴), 26.7 (C2) ppm; HRMS (ESI^+^) *m*/*z* calcd. for C_19_H_17_N_4_O_3_ [M + H^+^] 349.1295, found 349.1291.

1-(4-((1-(4-Fluorophenyl)-1*H*-1,2,3-triazol-4-yl)methoxy)-3,5-dimethoxyphenyl)ethan-1-one (**4a**). ~0.5 M Solution of 1-azido-4-fluorobenzene in *tert*-butyl methyl ether (1.8 mL, 0.896 mmol). The solid obtained was further purified through crystallization with acetone (0.128 g, 0.33 mmol, 53% yield). M.p. (acetone): 160.0–160.5 °C; IR (KBr) υ_max_: 3446, 3152, 3075, 2940, 2842, 1669, 1589, 1558, 1521, 1466, 1413, 1359, 1334, 1225, 1130, 964, 846, 612 cm^−1^; ^1^H NMR (CDCl_3_, 300.13 MHz) δ: 8.14 (1H, s, H-3″), 7.70 (2H, dd, *J* = 4.5 and 8.8 Hz, H-2‴, H-6‴), 7.25–7.20 (4H, m, H-3‴, H-5‴, H-2′, H-6′), 5.34 (2H, s, H-1″), 3.91 (6H, 3′,5′-OCH_3_), 2.59 (3H, s, H-2) ppm. ^13^C NMR (CDCl_3_, 75.47 MHz) δ: 197.1 (C1), 162.6 (d, *J* = 248.9 Hz, C4‴), 153.3 (C3′, C5′), 145.8 (C2″), 140.8 (C4′), 133.5 (C1‴), 133.2 (C1′), 122.7 (d, *J* = 8.7 Hz, C2‴, C6‴), 121.6 (C3″), 116.9 (d, *J* = 23.2 Hz, C3‴, C5‴), 105.8 (C2′, C6′), 66.6 (C1″), 56.5 (3′,5′-OCH_3_), 26.6 (C2) ppm; HRMS (ESI^+^) *m*/*z* calcd. for C_19_H_18_FN_3_NaO_4_ [M + Na^+^] 394.1174, found 394.1158.

1-(4-((1-(4-Fluorophenyl)-1*H*-1,2,3-triazol-4-yl)methoxy)-2-hydroxyphenyl)ethan-1-one (**4b**). ~0.5 M Solution of 1-azido-4-fluorobenzene in *tert*-butyl methyl ether (3.19 mL, 1.59 mmol). The obtained solid was purified through flash column chromatography (*n*-hexane:ethyl acetate, 8:2) (0.429 g, 1.31 mmol, 83% yield). M.p. (*n*-hexane:ethyl acetate): 145.9–148.0 °C; IR (KBr) υ_max_: 3129, 3079, 2961, 1923, 1884, 1629, 1518, 1458, 1253, 1231, 1066, 835, 810 cm^−1^; ^1^H NMR (CDCl_3_, 300.13 MHz) δ: 12.71 (1H, s, OH-2′), 8.03 (1H, s, H-3″), 7.74–7.65 (3H, m, H-6′, H-2‴, H-6‴), 7.26–7.20 (2H, m, H-3‴, H-5‴), 6.56–6.53 (2H, m, H-3′, H-5′), 5.31 (2H, s, H-1″), 2.56 (3H, s, H-2) ppm.; ^13^C NMR (CDCl_3_, 75.47 MHz) δ: 202.9 (C1), 165.2 (C2′), 164.6 (C4′), 162.7 (d, *J* = 249.8 Hz, C4‴), 144.1 (C2″), 133.2 (C1‴), 132.7 (C6′), 122.8 (d, *J* = 8.7 Hz, C2‴, C6‴), 121.4 (C3″), 117.0 (d, *J* = 23.3 Hz, C3‴, C5‴), 114.6 (C1′), 107.8 (C5′), 102.1 (C3′), 62.1 (C1″), 26.4 (C2) ppm; HRMS (ESI^+^) *m*/*z* calcd. for C_17_H_15_FN_3_O_3_ [M + H^+^] 328.1092, found 328.1095.

1-(4-((1-(4-Bromophenyl)-1*H*-1,2,3-triazol-4-yl)methoxy)-3,5-dimethoxyphenyl)ethan-1-one (**5a**). ~0.5 M Solution of 1-azido-4-bromo benzene in *tert*-butyl methyl ether (1.4 mL, 0.70 mmol). The solid obtained was further purified through crystallization with acetone (0.179 g, 0.41 mmol, 65% yield). M.p. (acetone): 159.1–160.4 °C; IR (KBr) υ_max_: 3447, 3167, 3098, 3005, 2942, 2838, 1671, 1591, 1498, 1465, 1456, 1416,1357, 1330, 1220, 1204, 1128, 1023, 986, 836, 607 cm^−1^; ^1^H NMR (CDCl_3_, 300.13 MHz) δ: 8.18 (1H, s, H-3″), 7.64 (4H, dd, *J* = 12.0, 9.0 Hz, H-2‴, H-3‴, H-5‴, H-6‴), 7.21 (2H, s, H-2′, H-6′), 5.34 (2H, s, H-1″), 3.91 (6H, s, 3′,5′-OCH_3_), 2.59 (3H, s, H-2) ppm; ^13^C NMR (CDCl_3_, 75.47 MHz) δ: 197.0 (C1), 153.3 (C3′, C5′), 146.0 (C2″), 140.8 (C4′), 136.2 (C1‴), 133.2 (C1′), 133.1 (C3‴, C5‴), 122.6 (C3″), 122.0 (C2‴, C6‴), 121.3, (C4‴), 105.8 (C2′, C6′), 66.5 (C1″), 56.4 (3′,5′-OCH_3_), 26.6 (C2) ppm; HRMS (ESI^+^) *m*/*z* calcd. for C_19_H_19_BrN_3_O_4_ [M + H^+^] 432.0553, found 432.0556.

1-(4-((1-(4-Bromophenyl)-1*H*-1,2,3-triazol-4-yl)methoxy)-2-hydroxyphenyl)ethan-1-one (**5b**). ~0.5 M Solution of 1-azido-4-bromobenzene in *tert*-butyl methyl ether (2.89 mL, 1.45 mmol). The obtained solid was purified through flash column chromatography (*n*-hexane:ethyl acetate, 8:2) (0.21 g, 0.54 mmol, 41% yield). M.p. (*n*-hexane:ethyl acetate): 183.3–184.8 °C; IR (KBr) υ_max_: 3138, 3101, 3068, 2920, 2865, 1647, 1618, 1585, 1267, 1169, 837, 783 cm^−1^; ^1^H NMR (DMSO-d_6_, 300.13 MHz) δ: 12.63 (1H, s, OH-2′), 9.00 (1H, s, H-3″), 7.90 (2H, d, *J* = 8.7 Hz, H-2‴, H-6‴), 7.87 (1H, d, *J* = 7.9 Hz, H-6′), 7.81 (2H, d, *J* = 8.9 Hz, H-3‴, H-5‴), 6.67–6.62 (2H, m, H-3′, H-5′), 5.33 (2H, s, H-1″), 2.57 (3H, s, H-2) ppm; ^13^C NMR (DMSO-d_6_, 75.47 MHz) δ: 203.3 (C1), 164.3, 164.0 (C2′, C4′), 143.4 (C2″), 135.8 (C1‴), 133.5 (C6′), 132.9 (C3‴, C5‴), 123.2 (C3″), 122.2 (C2‴, C6‴), 121.6 (C4‴), 114.1 (C1′), 107.7 (C5′), 101.7 (C3′), 61.4 (C1″), 26.7 (C2) ppm; HRMS (ESI^+^) *m*/*z* calcd. for C_17_H_15_BrN_3_O_3_ [M + H^+^] 388.0291, found 388.0292.

1-(4-((1-(4-Chlorophenyl)-1*H*-1,2,3-triazol-4-yl)methoxy)-3,5-dimethoxyphenyl)ethan-1-one (**6a**). ~0.5 M Solution of 1-azido-4-chloro benzene in *tert*-butyl methyl ether (1.4 mL, 0.70 mmol). The solid obtained was further purified through crystallization with acetone (0.12 g, 0.30 mmol, 47% yield). M.p. (acetone): 152.0–153.7 °C; IR (KBr) υ_max_: 3442, 3140, 2962, 2839, 1678, 1588, 1500, 1457, 1415, 1354, 1330, 1229, 1128, 956, 831, 608 cm^−1^; ^1^H NMR (CDCl_3_, 300.13 MHz) δ: 8.22 (1, s, H-3″), 7.69 (2H, d, *J* = 8.8 Hz, H-2‴, H-6‴), 7.51 (2H, d, *J* = 8.7 Hz, H-3‴, H-5‴), 7.22 (2H, s, H-2′, H-6′), 5.34 (2H, s, H-1″), 3.91 (6H, s, 3′,5′-OCH_3_), 2.59 (3H, s, H-2) ppm; ^13^C NMR (CDCl_3_, 75.47 MHz) δ: 197.0 (C1), 153.3 (C3′, C5′), 140.8 (C4′), 134.7 (C2″), 133.2 (C1‴), 130.2 (C4‴), 121.9 (C3″, C2‴, C6‴), 105.8 (C2′, C6′), 66.4 (C1″), 56.4 (3′,5′-OCH_3_), 26.6 (C2) ppm; HRMS (ESI^+^) *m*/*z* calcd. for C_19_H_19_ClN_3_O_4_ [M + H^+^] 388.1059, found 388.1055.

1-(4-((1-(4-Chlorophenyl)-1*H*-1,2,3-triazol-4-yl)methoxy)-2-hydroxyphenyl)ethan-1-one (**6b**). ~0.5 M Solution of 1-azido-4-chlorobenzene in *tert*-butyl methyl ether (3.19 mL, 1.59 mmol). The obtained solid was purified through flash column chromatography (*n*-hexane:ethyl acetate, 8:2) (0.277 g, 0.81 mmol, 61% yield). M.p. (*n*-hexane:ethyl acetate): 157.0–159.1 °C; IR (KBr) υ_max_: 3445, 3139, 3102, 3070, 1651, 1621, 1504, 1369, 1348, 1266, 840, 783 cm^−1^; ^1^H NMR (CDCl_3_, 300.13 MHz) δ: 12.71 (1H, s, OH-2′), 8.05 (1H, s, H-3″), 7.70 (2H, d, *J* = 8.8 Hz, H-2‴, H-6‴), 7.66 (1H, d, *J* = 9.7 Hz, H-6′), 7.51 (2H, d, *J* = 8.7 Hz, H-3‴, H-5‴), 6.57–6.53 (2H, m, H-3′, H-5′), 5.31 (2H, s, H-1″), 2.56 (3H, s, H-2) ppm; ^13^C NMR (CDCl_3_, 75.47 MHz) δ: 202.9 (C1), 165.2 (C2′), 164.4 (C4′), 144.3 (C2″), 135.5 (C1‴), 135.0 (C4‴), 132.7 (C6′), 130.2 (C3‴, C5‴), 121.9 (C2‴, C6‴), 121.2 (C3″), 114.6 (C1′), 107.8 (C5′), 102.2 (C3′), 62.1 (C1″), 26.4 (C2) ppm; HRMS (ESI^+^) *m*/*z* calcd. for C_17_H_15_ClN_3_O_3_ [M + H^+^] 344.0796, found 344.0797.

1-(4-((1-(3-Hydroxypropyl)-1*H*-1,2,3-triazol-4-yl)methoxy)-3,5-dimethoxyphenyl)ethan-1-one (**7a**). 3-Azido-1-propanol (0.66 mL, 0.768 mmol); The solid obtained was further purified through crystallization with ethyl acetate and flash column chromatography (chloroform:acetone, 8:2) (0.120 g, 0.357 mmol, 56% yield). M.p. (chloroform:acetone): 90.0–93.0 °C; IR (KBr) υ_max_: 3532, 3137, 3008, 2947, 2927, 2869, 2839, 2790, 1680, 1592, 1506, 1466, 1457, 1414, 1362, 1328, 1182, 1128, 1058, 954, 843, 816, 653, 609 cm^−1^; ^1^H NMR (CDCl_3_, 300.13 MHz) *δ*: 7.78 (1H, s, H-3″), 7.19 (H-2′, H-6′), 5.28 (2H, s, H-1″), 4.53 (2H, brt, H-1‴), 3.90 (6H, s, 3′,5′-OCH_3_), 3.62 (2H, t, *J* = 5.1 Hz, H-3‴), 2.58 (3H, s, H-2), 2.12 (2H, brt, H-2″) ppm; ^13^C NMR (CDCl_3_, 75.47 MHz) *δ*: 197.1 (C1), 153.3 (C3′, C5′), 140.8 (C4′, C2″), 133.1 (C1′, C3″), 105.8 (C2′, C6′), 66.5 (C1″), 58.9 (C3″), 56.5 (3′,5′-OCH_3_), 47.2 (C1″), 32.7 (C2″), 26.6 (C2) ppm; HRMS (ESI^+^) *m*/*z* calcd. for C_16_H_22_N_3_O_5_ [M + H^+^] 336.1554, found 336.1552.

1-(2-Hydroxy-4-((1-(3-hydroxypropyl)-1*H*-1,2,3-triazol-4-yl)methoxy)phenyl)ethan-1-one (**7b**). 3-Azido-1-propanol (0.049 mL, 0.53 mmol); The obtained solid was purified through flash column chromatography (chloroform:methanol, 98:2). (0.061 g, 0.21 mmol, 40% yield). M.p. (chloroform:methanol): 69.2–70.5 °C; IR (KBr) υ_max_: 3311, 3133, 3079, 2922, 2876, 1636, 1615, 1576, 1505, 1270, 1054, 1037, 985, 959, 836, 800 cm^−1^; ^1^H NMR (DMSO-d_6_, 300.13 MHz) δ: 8.25 (1H, s, H-3″), 7.85 (1H, d, *J* = 8.7 Hz, H-6′), 6.62 (1H, d, *J* = 2.4 Hz, H-3′), 6.59 (1H, dd, *J* = 8.7, 2.5 Hz, H-5′), 5.21 (2H, s, H-1″), 4.43 (1H, t, *J* = 7.1 Hz, H-1‴), 3.42–3.38 (signal under water, H-3‴), 2.57 (3H, s, H-2), 2.00–1.92 (2H, m, H-2‴) ppm; ^13^C NMR (DMSO-d_6_, 75.47 MHz) δ: 203.2 (C1), 169.2, 164.4 (C4), 164.1 (C2), 141.9 (C2″), 133.4 (C6′), 124.9 (C3″), 114.0 (C1′), 107.7 (C5′), 101.6 (C3′), 61.5 (C1″), 57.9 (C3‴), 46.8 (C1‴), 32.9 (C2‴), 26.7 (C2) ppm; HRMS (ESI^+^) *m*/*z* calcd. for C_14_H_18_N_3_O_4_ [M + H^+^] 292.1292, found 292.1298.

1-(3,5-Dimethoxy-4-((1-(4-methoxyphenyl)-1*H*-1,2,3-triazol-4-yl)methoxy)phenyl)ethan-1-one (**8a**). The solid obtained was further purified through flash column chromatography (*n*-hexane:ethyl acetate, 8:2 and 7:3) (0.074 g, 0.192 mmol, 30% yield). M.p. (*n*-hexane:ethyl acetate): 99.5–102.3 °C; IR (KBr) υ_max_: 3432, 3153, 3090, 2999, 2934, 2839, 1670, 1585, 1522, 1462, 1412, 1330, 1258, 1216, 1182, 1130, 1033, 982, 839, 827, 811, 652, 609 cm^−1^; ^1^H NMR (CDCl_3_, 300.13 MHz) δ: 8.06 (1H, s, H-3″), 7.61 (2H, dd, *J* = 6.8, 2.1 Hz, H-2‴, H-6‴), 7.21 (2H, s, H-2′, H-6′), 7.02 (2H, dd, *J* = 6.7, 2.2 Hz, H-3‴, H-5‴), 5.34 (2H, s, H-1″), 3.90 (6H, s, 3′,5′-OCH_3_), 3.87 (3H, s, 4‴-OCH_3_), 2.59 (3H, s, H-2) ppm; ^13^C NMR (CDCl_3_, 75.47 MHz) δ: 197.1 (C1), 160.0 (C4‴), 153.4 (C3′, C5′), 145.3 (C2″), 140.9 (C4′), 133.1 (C1′), 130.7 (C1‴), 122.3 (C2‴, C6‴), 121.5 (C3″), 114.9 (C-3‴, C5‴), 105.8 (C2′, C6′), 66.6 (C1″), 57.0 (3′,5′-OCH_3_), 55.8 (4‴-OCH_3_), 25.6 (C2) ppm; HRMS (ESI^+^) *m*/*z* calcd. for C_20_H_22_N_3_O_5_ [M + H^+^] 384.1554, found 384.1551.

1-(2-Hydroxy-4-((1-(4-methoxyphenyl)-1*H*-1,2,3-triazol-4-yl)methoxy)phenyl)ethan-1-one (**8b**). ~0.5 M Solution of 4-azidoanisole in *tert*-butyl methyl ether (2.66 mL, 1.33 mmol). The obtained solid was purified through flash column chromatography (*n*-hexane:ethyl acetate, 9:1) (0.179 g, 0.527 mmol, 40% yield). M.p. (*n*-hexane:ethyl acetate): 115.1–117.5 °C; IR (KBr) υ_max_: 3448, 3134, 3097, 2922, 1645, 1444, 1307, 1187, 861, 837, 812 cm^−1^; ^1^H NMR (CDCl_3_, 300.13 MHz) δ: 12.71 (1H, s, OH-2′), 7.99 (1H, s, H-3″), 7.66 (1H, d, *J* = 10.3 Hz, H-6′), 7.63 (2H, d, *J* = 9.1 Hz, H-2‴, H-6‴), 7.02 (2H, d, *J* = 9.0 Hz, H-3‴, H-5‴), 6.57–6.53 (2H, m, H-3′, H-5′), 5.30 (2H, s, H-1″), 3.87 (3H, s, 4‴-OCH_3_), 2.56 (3H, s, H-2) ppm; ^13^C NMR (CDCl_3_, 75.47 MHz) δ: 202.8 (C1), 165.2 (C2′), 164.7 (C4′), 160.1 (C4‴), 143.7 (C2″), 132.7 (C6′), 130.4 (C1‴), 122.4 (C2‴, C6‴), 121.5 (C3″), 115.0 (C3‴, C5‴), 114.5 (C1′), 107.8 (C5′), 102.2 (C3′), 62.2 (C1″), 26.4 (C2) ppm; HRMS (ESI^+^) *m*/*z* calcd. for C_18_H_18_N_3_O_4_ [M + H^+^] 340.1292, found 340.1294.

*N*-((2*S*,3*S*,4*S*,5*R*,6*S*)-2-(4-((4-Acetyl-2,6-dimethoxyphenoxy)methyl)-1*H*-1,2,3-triazol-1-yl)-4,5-dihydroxy-6-(hydroxymethyl)tetrahydro-2*H*-pyran-3-yl)acetamide (**9a**). 2-Azidoethyl 2-acetamido-2-deoxy-*β*-d-glucopyranosyl azide (0.73 g, 3.0 mmol). A solid formed in the aqueous phase was filtered and washed with methanol. The solid was further purified by crystallization with methanol. Whitish solid (0.38 g, 0.79 mmol, 54% yield). dec pt: 200 °C; IR (KBr) υ_max_: 3354, 3126, 2960, 2879, 1680, 1646, 1589, 1551, 1466, 1330, 1202, 1178, 890 cm^−1^; ^1^H NMR (DMSO-d_6_, 300.13 MHz) *δ*: 8.17 (1H, s, H-3″), 7.89 (1H, d, *J* = 9.2 Hz, 2‴-NH), 7.24 (2H, s, H-2′, H-6′), 5.72 (1H, d, *J* = 10.0 Hz, H-1‴), 5.29 (1H, d, *J* = 5.3 Hz, OH-sugar), 5.25 (1H, *J* = 5.5 Hz, OH-sugar), 5.04 (2H, d, *J* = 5.2 Hz, H-1″), 4.66 (1H, t, *J* = 5.7 Hz, OH-sugar), 4.08 (1H, q, *J* = 9.8 Hz, H-2‴), 3.85 (6H, s, 3′,5′-OCH_3_), 3.71 (1H, m, H-6‴), 3.59–3.28 (4H, m, H-sugar), 2.58 (3H, s, H-2), 1.62 (3H, s, 2‴-COCH_3_) ppm; ^13^C NMR (DMSO-d_6_, 75.47 MHz) *δ*: 196.6 (C1), 168.9 (2‴-COCH_3_), 152.6 (C3′, C5′), 142.8 (C2″), 139.9 (C4′), 132.2 (C1′), 122.8 (C3″), 105.5 (C2′, C6′), 85.7 (C1‴), 79.8 (C-sugar), 73.7 (C-sugar), 69.6 (C-sugar), 65.0 (C1″), 60.5 (C6‴), 55.8 (3′,5′-OCH_3_), 54.1 (C2‴), 26.4 (C2), 22.5 (2‴-COCH_3_) ppm; HRMS (ESI-TOF) *m*/*z* calcd. for C_21_H_28_N_4_O_4_Na 503.17485, found 503.17612.

*N*-((2*R*,3*R*,4*R*,5*S*,6*R*)-2-(4-((4-Acetyl-3-hydroxyphenoxy)methyl)-1*H*-1,2,3-triazol-1-yl)-4,5-dihydroxy-6-(hydroxymethyl)tetrahydro-2*H*-pyran-3-yl)acetamide (**9b**). 2-Azidoethyl 2-acetamido-2-deoxy-β-d-glucopyranosyl azide (0.142 g, 0.578 mmol). A solid formed in the aqueous phase was separated by filtration and washed with water. The solid was further purified by crystallization with ethanol (0.080 g, 0.183 mmol, 35% yield); dec pt: 221 °C; IR (KBr) υ_max_: 3320, 3089, 2908, 2850, 1655, 1620, 1522, 1375, 1073, 1057, 1047, 1030, 825, 798 cm^−1^; ^1^H NMR (DMSO-d_6_, 300.13 MHz) δ: 12.62 (1H, s, OH-2′), 8.30 (1H, s, H-3″), 7.88 (1H, d, *J* = 9.4 Hz, 2‴-NH), 7.85 (1H, d, *J* = 9.2 Hz, H-6′), 6.61–6.58 (2H, m, H-3′, H-5′), 5.73 (1H, d, *J* = 10.0 Hz, H-1‴), 5.21 (2H, s, H-1″), 5.30–5.25 (2H, m, OH-sugar), 4.66 (1H, t, *J* = 6.5 Hz, OH-sugar), 4.07 (1H, q, *J* = 9.7 Hz, H-2‴), 3.73–3.68 (1H, m, H-sugar), 3.59–3.28 (m, H-sugar), 2.57 (3H, s, H-2), 1.61 (3H, s, 2‴-COCH_3_) ppm; ^13^C NMR (DMSO-d_6_, 75.47 MHz) δ: 203.3 (C1), 169.2 (2‴-COCH_3_), 164.4 (C4), 164.0 (C2), 141.8 (C2″), 133.4 (C6′), 123.6 (C3″), 114.0 (C1′), 107.7 (C5′), 101.7 (C3′), 86.1 (C1‴), 80.1 (C-sugar), 74.0 (C-sugar), 69.9 (C-sugar), 61.4 (C1″), 60.7 (C6‴), 54.5 (C2‴), 26.7 (C2), 22.7 (2‴-COCH_3_) ppm; HRMS (ESI^+^) *m*/*z* calcd. for C_19_H_25_N_4_O_8_ [M + H^+^] 437.1667, found 437.1669.

### 3.3. Mussel (Mytilus galloprovincialis) Larvae Anti-Settlement Activity

Mussel (*Mytilus galloprovincialis*) plantigrades were collected in juvenile aggregates during low neap tides at Memória beach, Matosinhos, Portugal (41°13′59″ N; 8°43′28″ W). In the laboratory, mussel plantigrade larvae (0.5–2 mm) were isolated in a binocular magnifier (Olympus SZX2-ILLT, Tokyo, Japan) to a petri dish with filtered seawater, and those with functional foot and competent exploring behavior were selected to the bioassays. Compounds were screened at 50 µM in 24-well microplates with 4-well replicates per condition and 5 larvae per well, for 15 h, in the darkness at 18 ± 1 °C, according to the previously reported [35,36]. All compounds that caused more than 60% of settlement inhibition (≤40% of settlement) in the screening bioassay were considered active and selected for the determination of the semi-maximum response concentration that inhibited 50% of the larval settlement (EC_50_), at compounds concentrations of 3.125, 6.25, 12.5, 25, 50, 100, and 200 μM.

### 3.4. Biofilm-Forming Marine Bacteria Growth Inhibitory Activity

Five strains of marine biofilm-forming bacteria from the Spanish Type Culture Collection (CECT): *Cobetia marina* CECT 4278, *Vibrio harveyi* CECT 525, *Halomonas aquamarina* CECT 5000, *Pseudoalteromonas atlantica* CECT 570, and *Roseobacter litoralis* CECT 5395 were selected for antibacterial screening. The experimental procedure was performed according to the previously reported [35,36]. Briefly, bacteria were inoculated and incubated for 24 h at 26 °C in marine broth (Difco) at an initial density of 0.1 (OD600) in 96-well flat-bottom microtiter plates and exposed to the test compounds at 15 μM. Bacterial growth inhibition in the presence of the compounds was determined in quadruplicate at 600 nm using a microplate reader (BioTek Synergy HT, Winooski, VT, USA). Negative and positive controls used were a solution of marine broth with 0.1% DMSO, and a solution of marine broth with penicillin–streptomycin–neomycin, respectively. Bacterial growth inhibition calculations were made based in the formula ((Mc–Mt)/Mc) × 100, where Mc is the mean of the four replicates from negative control, and Mt is the mean of four replicates from each of the tested compounds.

### 3.5. Antifungal Susceptibility Testing

The antifungal activity was evaluated against *Candida albicans* ATCC 10231, *Aspergillus fumigatus* ATCC 204305, and *Trichophyton rubrum*-FF5. *Candida krusei* ATCC 6258 was used for quality control. To guarantee the purity and viability, the strains were sub-cultured before each assay on Sabouraud dextrose agar (BioMérieux, Marcy l’Etoile, France). RPMI-1640 broth medium pH 7.0, with L-glutamine and without bicarbonate (Biochrom) and buffered with 0.165 mol·L^−1^ of 3-(*N*-morpholino)-propanesulfonic acid (MOPS, Sigma-Aldrich, St. Louis, MO, USA), was used on the evaluation of the antifungal activity. The MICs were evaluated using the broth microdilution method and in accordance with the recommendations of the Clinical and Laboratory Standards Institute (CLSI) reference documents: M27-A3 for yeasts [37] and M38-A2 [38] for filamentous fungi. Two-fold serial dilutions of compounds were prepared within the concentration range of 8–128 µg·mL^−1^. Yeast cells suspensions were prepared to obtain an inoculum of 1–5 × 10^3^ CFU·mL^−1^. For filamentous fungi, a spore suspension is prepared, and cell density was adjusted to obtain the adequate inoculum (for dermatophytes 1–3 × 10^3^ CFU·mL^−1^ and 0.4–5 × 10^4^ CFU·mL^−1^ for *Aspergillus fumigatus*). Equal volumes of compound dilution in RPMI and cell suspension in RPMI were added in the wells of the microplate. Controls performed were sterility control, growth control, and quality control. Quality control was performed with an ATCC reference strain (*Candida krusei* ATCC 6258) with a commercial antifungal compound, voriconazole (Pfizer) ranging between 0.25–1 µg·mL^−1^. The plates were incubated aerobically at 35 °C for 48 h for *Candida albicans* and A*spergillus fumigatus* and at 25 °C for 5–7 days for dermatophytes. MICs were determined as the lowest concentrations resulting in 100% growth inhibition, in comparison to the compound-free controls. All the compounds were tested independently three times.

### 3.6. Biofilm-Forming Marine Diatoms Growth Inhibitory Activity

The anti-microalgal activity of the most promising compounds was also evaluated against a benthic marine diatom, *Navicula* sp., purchased from the Spanish Collection of Algae (BEA), according to the previously reported [16]. Briefly, diatom cells were inoculated in f/2 medium (Sigma) at an initial concentration of 2–4 × 10^6^ cells·mL^−1^ and grown in 96-well flat-bottom microtiter plates for 14 days in continuous light at 20 °C. *Navicula* sp. growth inhibition in the presence of each compound at 25 μM was determined in quadruplicate, and cells were counted using a Neubauer counting chamber. Growth inhibition was calculated based in the formula ((Mc–Mt)/Mc) × 100, where Mc is the mean of the cell counts of the four replicates from negative control, and Mt is the mean of the cell counts of the four replicates from each of the tested compounds. Positive control with cycloheximide (3.55 μM) and negative control with f/2 medium 0.1% DMSO were included.

### 3.7. Artemia Salina Ecotoxicity Bioassay

The brine shrimp (*Artemia salina*) nauplii lethality test was used to determine the ecotoxicity of **6a**, **7a**, and **9a** to non-target organisms [35]. Briefly, *Artemia salina* eggs were allowed to hatch in seawater for 48 h at 25 °C. Bioassays were performed in 96-well microplates with 15–20 nauplii per well and 200 μL of the compounds test solution. Test solutions were prepared in filtered seawater at concentrations of 25 and 50 μM. All tests included K_2_Cr_2_O_7_ as positive control and DMSO as a negative control. Bioassays run in the dark at 25 °C, and the percentage of mortality was determined after 48 h of exposure.

### 3.8. In Silico Evaluation of LogKow

Compounds are considered potentially bioaccumulative if the LogKow (octanol–water partition coefficient) is higher than 3. Therefore, the LogKow value is used as an indicator of the bioaccumulation potential of AF compounds. KOWWIN™ v1.68 (a Log octanol–water partition coefficient calculation program), developed by the United States Environmental Protection Agency (EPA) and the Syracuse Research Corporation (SRC) [39] was used for the in silico calculation of LogKow of the most active compounds in this study.

### 3.9. Statistical Analysis

Data from anti-settlement, antibacterial, and anti-microalgal bioassay were analyzed by one-way analysis of variance (ANOVA), followed by a multi-comparisons Dunnett’s test against negative control. For all of the bioassays, the half-maximum response concentration (EC_50_) values for each compound, when applicable, were calculated using probit regression analysis. Significance was considered at *p* < 0.01, and 95% lower and upper confidence limits (95% LCL; UCL). The software IBM SPSS Statistics 26 (Armonk, New York, NY, USA) was used for statistical analysis.

## 4. Conclusions

Considering the biological potential of phenyl ketones and 1,2,3-triazole ring, in this work 14 acetophenone-1,2,3-triazole hybrids containing different substitution patterns were synthesized and tested for their AF activity in both macro- and microfouling species. Among them, three compounds (**6a**, **7a**, and **9a**), containing methoxy groups in the phenyl ketone core with different substituents linked to the heterocyclic ring, revealed to be the most promising compounds against mussel larvae, with EC_50_ values lower than 25 µg·mL^−1^, while acetophenones **3b**, **4b**, and **7b** showed some inhibitory effect against the growth of biofilm-forming bacteria *Roseobacter litoralis*. In addition to the activity on macrofouling species, compound **7a** also showed AF activity against the microalgae *Navicula* sp. (EC_50_ = 26.73 µM, 8.96 µg·mL^−1^), suggesting a complementary action of this compound against macro- and microfouling species. The most promising compounds of this study (**7a** and **9a**) were also shown to be non-toxic against the non-target species *Artemia salina*, as well as low bioaccumulative potential. The overall results highlight **7a** and **9a** as promising compounds, which could be considered hits for the development of effective and eco-friendly AF compounds.

## Figures and Tables

**Table 1:** Synthesis of acetophenone hybrids **3a**–**9b**.

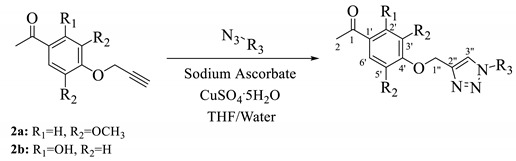				
Compound	R_1_	R_2_	R_3_	Yield (%)
**3a**	H	OCH_3_	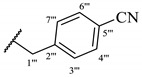	65
**3b**	OH	H		84
**4a**	H	OCH_3_	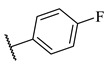	53
**4b**	OH	H		83
**5a**	H	OCH_3_	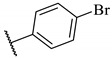	65
**5b**	OH	H		41
**6a**	H	OCH_3_	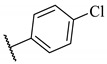	47
**6b**	OH	H		61
**7a**	H	OCH_3_	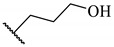	56
**7b**	OH	H		40
**8a**	H	OCH_3_	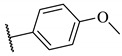	30
**8b**	OH	H		40
**9a**	H	OCH_3_	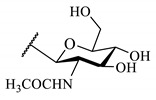	54
**9b**	OH	H		35

**Table 2:** Anti-settlement effectiveness and toxicity parameters of derivatives **6a**, **7a**, and **9a** towards mussel plantigrade larvae.

Compounds	EC_50_ (µM)	EC_50_ (µg·mL^−1^)	LC_50_ (µM)	LC_50_/EC_50_
**6a**	28.87 (95% CI: 18.93–44.39)	11.20	>200	>6.93
**7a**	40.14 (95% CI: 23.95–69.63)	13.46	>200	>4.98
**9a**	20.68 (95% CI: 9.70–40.75)	9.94	>200	>9.67

EC_50_: minimum concentration that inhibited 50% of larval settlement; LC_50_: median lethal dose; LC_50_/EC_50_: therapeutic ratio; CI: confidence interval.

## Data Availability

Data is contained within the article.

## Supplementary Materials

The following are available online at www.mdpi.com/1660-3397/19/12/682/s1, Figures S1–S28: ^1^H NMR, ^13^C NMR and HRMS spectra of compounds **3a**–**9b**.
