# Supplementary material for: Natural Benzo/Acetophenones as Leads for New Synthetic Acetophenone Hybrids Containing a 1,2,3-Triazole Ring as Potential Antifouling Agents
Source: Mar Drugs. 2021 Nov 29;19(12):682. doi: 10.3390/md19120682 (PMC8704891; doi:10.3390/md19120682)
Supplement: Supplementary file 1 [file marinedrugs-19-00682-s001.zip › marinedrugs-1463739-supplementary.pdf]

Supplementary Material

Natural benzo/acetophenones as leads for new synthetic acetophenone hybrids containing a 1,2,3-triazole ring as potential antifouling agents

Ana Rita Neves [1,2,†], Daniela Pereira [1,2,†], Catarina Gonçalves [2], Joana Cardoso[1], Eugénia Pinto [2,3], Vitor Vasconcelos [2,4], Madalena Pinto [1,2], Emília Sousa [1,2], Joana R. Almeida [2*], Honorina Cidade [1,2*], Marta Correia-da-Silva [1,2]

1 Laboratory of Organic and Pharmaceutical Chemistry, Department of Chemical Sciences, Faculty of Pharmacy, University of Porto, Rua Jorge Viterbo Ferreira, 228, 4050-313, Porto, Portugal; anarcneves92@gmail.com (A.R.N.); dmpereira@ff.up.pt (D.P.); up201603353@edu.ff.up.pt (J.C); madalena@ff.up.pt (M.P.); esousa@ff.up.pt (E.S); m_correiadasilva@ff.up.pt (M.C.S.).
2 CIIMAR—Interdisciplinary Centre of Marine and Environmental Research, University of Porto, Avenida General Norton de Matos, 4450-208 Matosinhos, Portugal; catarina.goncalves@ciimar.up.pt (C.G.); epinto@ff.up.pt (E.P.); vmvascon@fc.up.pt (V.M.V.).
3 Laboratory of Microbiology, Department of Biological Sciences, Faculty of Pharmacy, University of Porto, Rua de Jorge Viterbo Ferreira, 228, 4050-313 Porto, Portugal.
4 Department of Biology, Faculty of Sciences, University of Porto, Rua do Campo Alegre, 4069-007 Porto, Portugal.

† These authors contributed equally to this work.

* Correspondence: jalmeida@ciimar.up.pt (J.R.A); hcidade@ff.up.pt (H.C.).

Table of contents

Figure S1 - ^{1}H NMR and ^{13}C NMR spectra of compound **3a**...3

Figure S2 - HRMS spectrum of compound **3a**. 4

Figure S3 - ^{1}H NMR and ^{13}C NMR spectra of compound **3b**. 5

Figure S4 - HRMS spectrum of compound **3b**...6

Figure S5 - ^{1}H NMR and ^{13}C NMR spectra of compound **4a**...7

Figure S6 - HRMS spectrum of compound **4a**. 8

Figure S7 - ^{1}H NMR and ^{13}C NMR spectra of compound **4b**. 9

Figure S8 - HRMS spectrum of compound **4b**. 10
Figure S9 - ^{1}H NMR and ^{13}C NMR spectra of compound **5a** 11
Figure S10 - HRMS spectrum of compound **5a**. 12
Figure S11 - ^{1}H NMR and ^{13}C NMR spectra of compound **5b**. 13
Figure S12 - HRMS spectrum of compound **5b**. 14
Figure S13 - ^{1}H NMR and ^{13}C NMR spectra of compound **6a** 15
Figure S14 - HRMS spectrum of compound **6a**. 16
Figure S15 - ^{1}H NMR and ^{13}C NMR spectra of compound **6b**. 17
Figure S16 - HRMS spectrum of compound **6b**. 18
Figure S17 - ^{1}H NMR and ^{13}C NMR spectra of compound **7a** 19
Figure S18 - HRMS spectrum of compound **7a**. 20
Figure S19 - ^{1}H NMR and ^{13}C NMR spectra of compound **7b**. 21
Figure S20 - HRMS spectrum of compound **7b**. 22
Figure S21 - ^{1}H NMR and ^{13}C NMR spectra of compound **8a** 23
Figure S22 - HRMS spectrum of compound **8a**. 24
Figure S23 - ^{1}H NMR and ^{13}C NMR spectra of compound **8b**. 25
Figure S24 - HRMS spectrum of compound **8b**. 26
Figure S25 - ^{1}H NMR and ^{13}C NMR spectra of compound **9a** 27
Figure S26 - HRMS spectrum of compound **9a**. 28
Figure S27 - ^{1}H NMR and ^{13}C NMR spectra of compound **9b**. 29
Figure S28 - HRMS spectrum of compound **9b**. 30

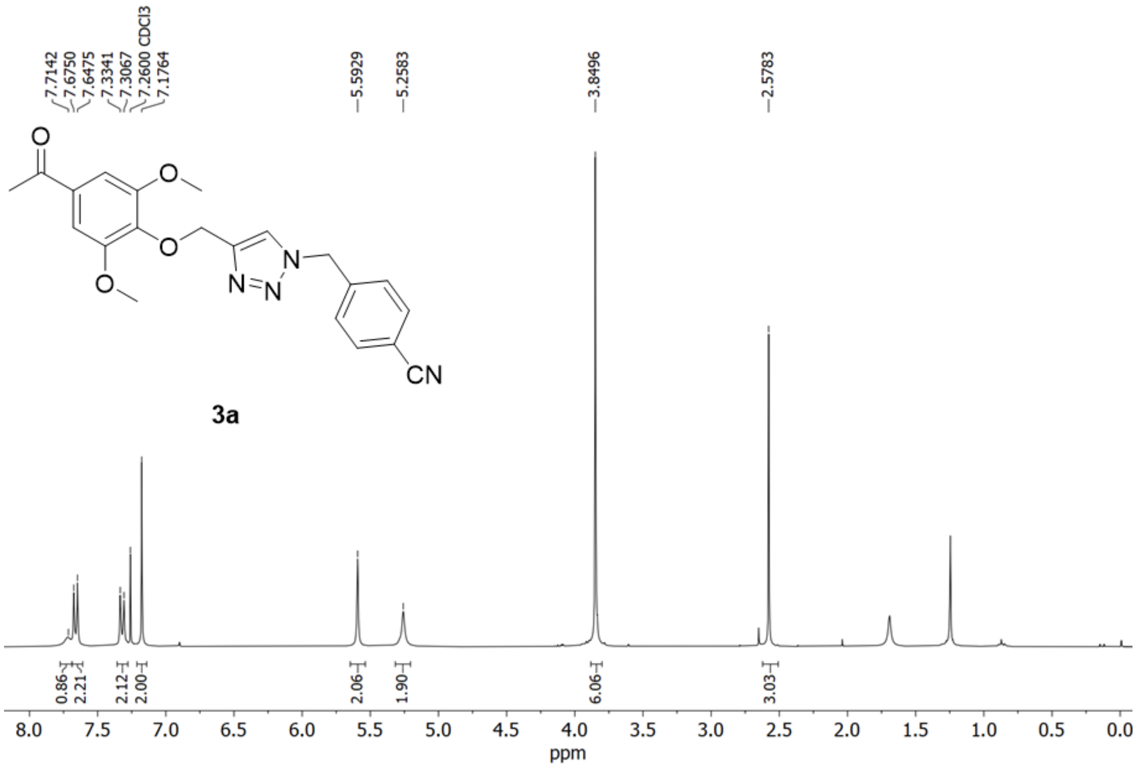

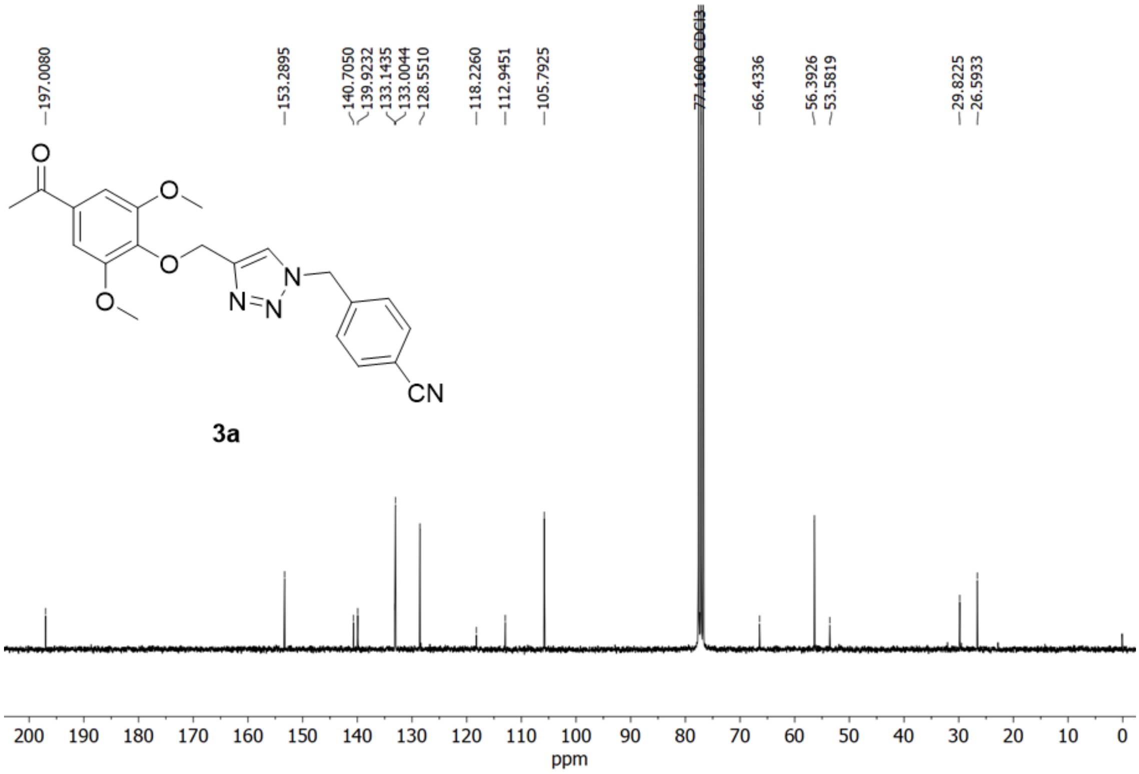

Figure S1 - ^{1}H NMR and ^{13}C NMR spectra of compound **3a**.

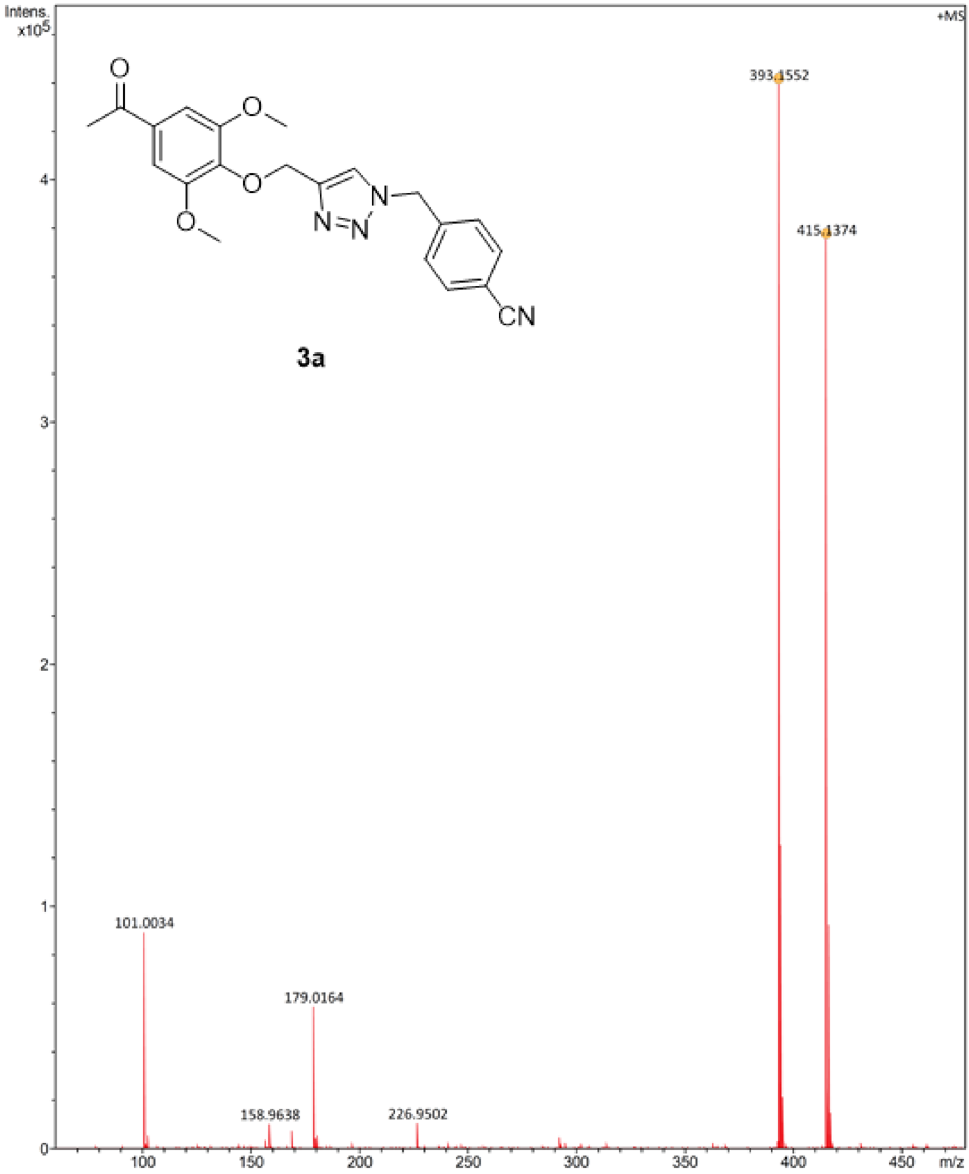

Meas. m/z	Formula	m/z	err [ppm]
393.1552	$C_{21}H_{21}N_4O_4$	393.1557	1.4

Figure S2 - HRMS spectrum of compound **3a**.

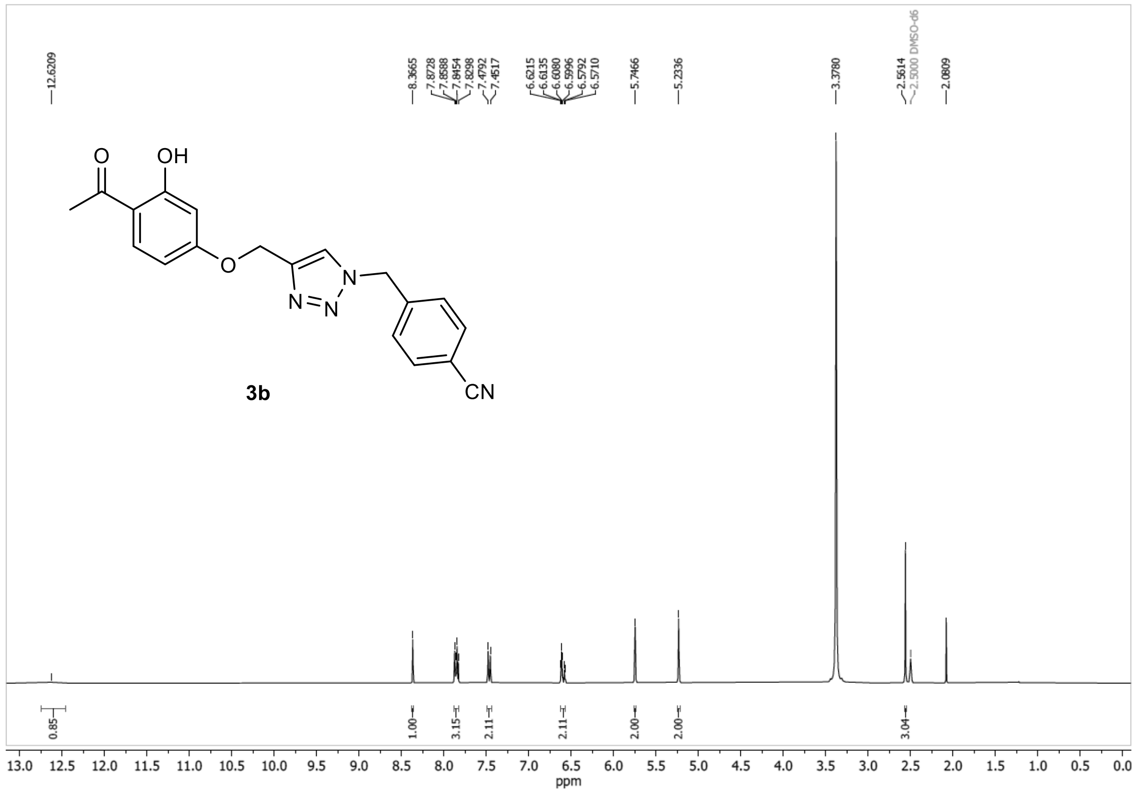

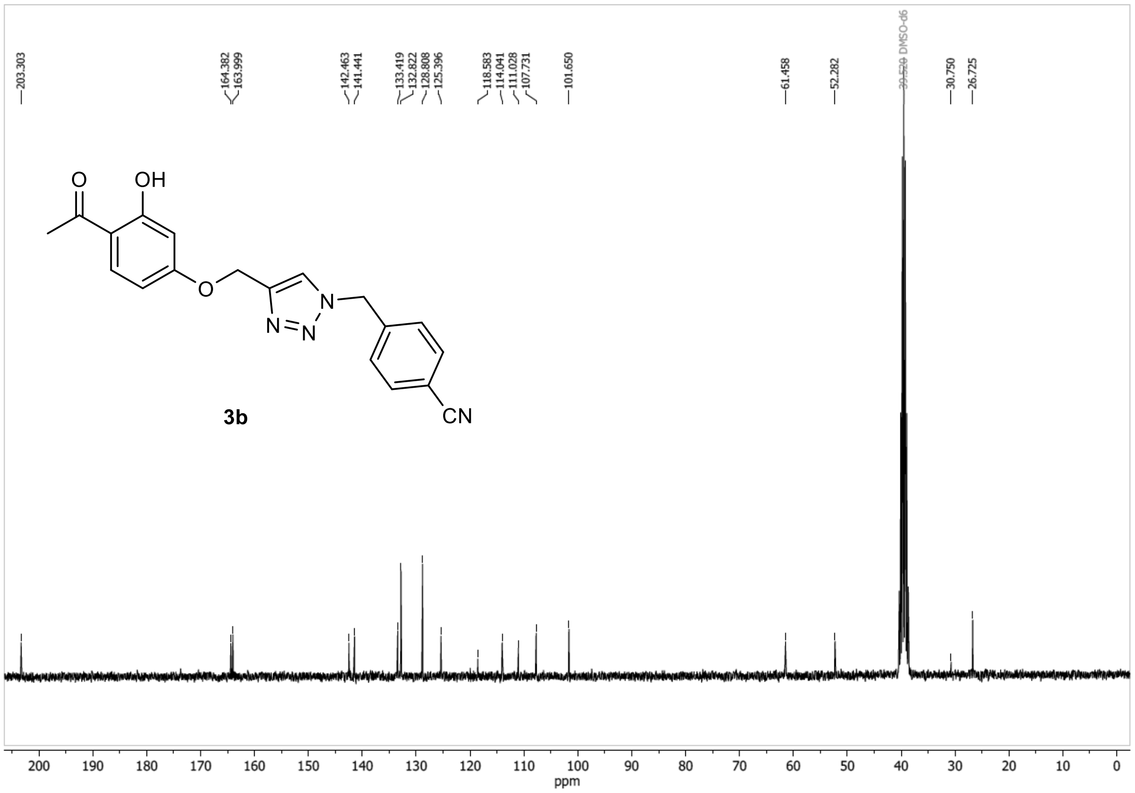

Figure S3 - ^{1}H NMR and ^{13}C NMR spectra of compound **3b**.

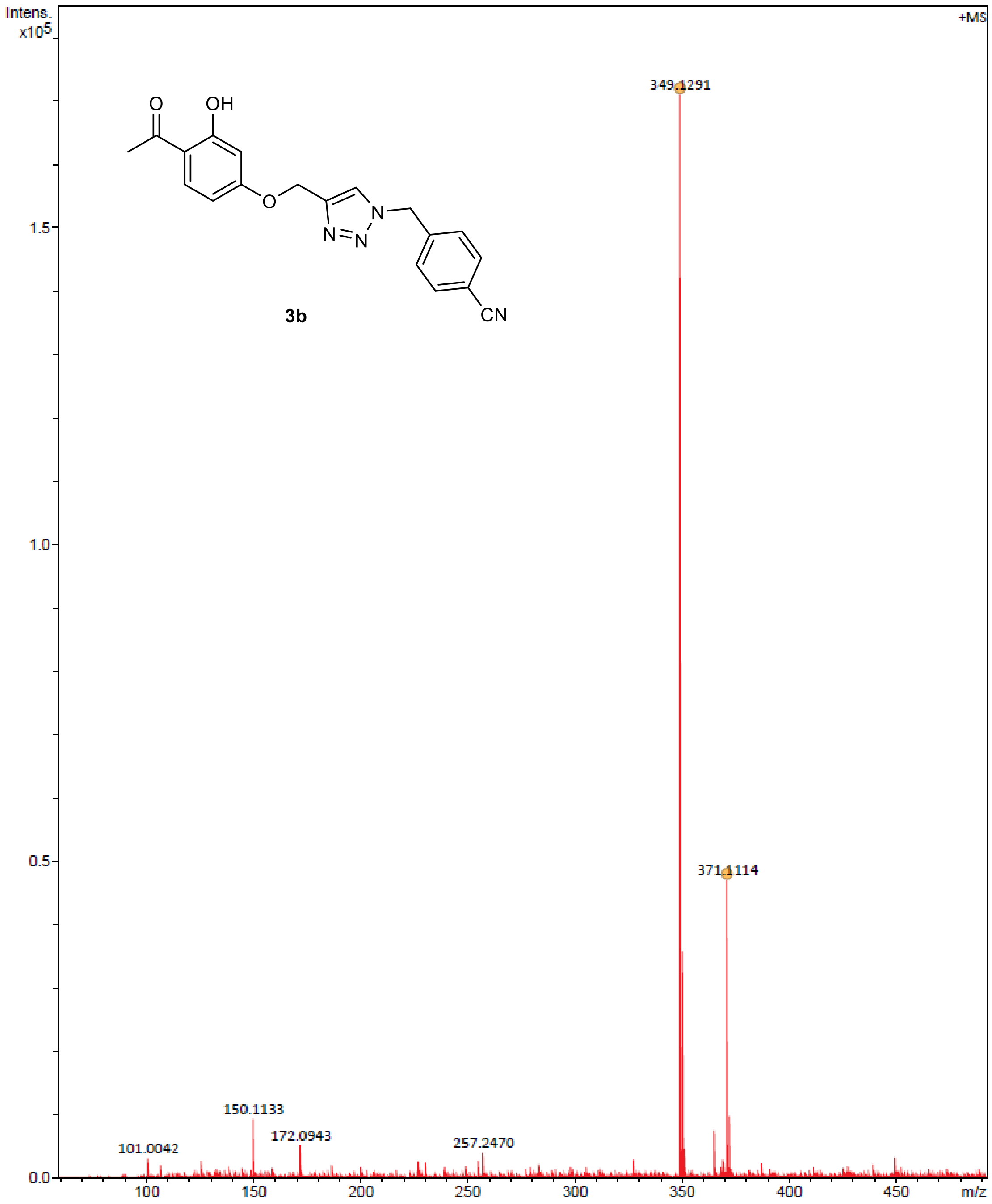

Mass Spectrum Molecular Formula Report

Meas. m/z	#	Ion Formula	m/z	err [ppm]	mSigma	# mSigma	Score	rdb	e⁻ Conf	N-Rule
349.1291	1	C19H17N4O3	349.1295	1.2	8.5	1	100.00	13.5	even	ok

Figure S4 - HRMS spectrum of compound **3b**.

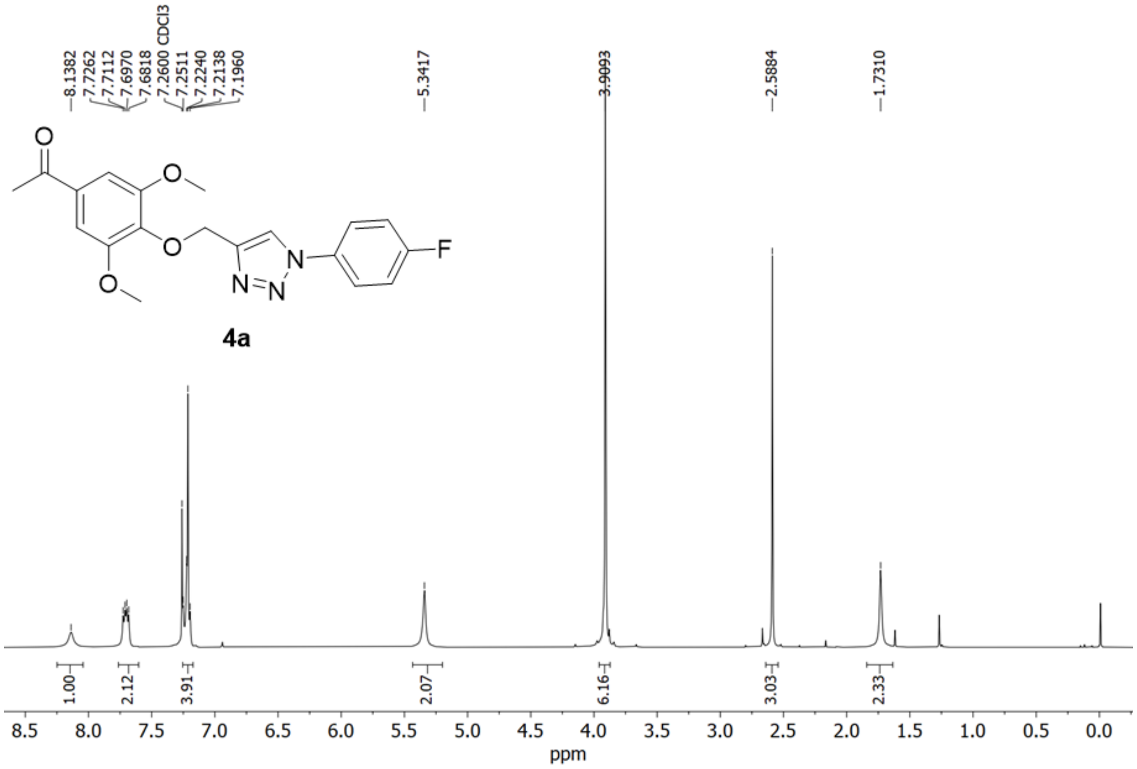

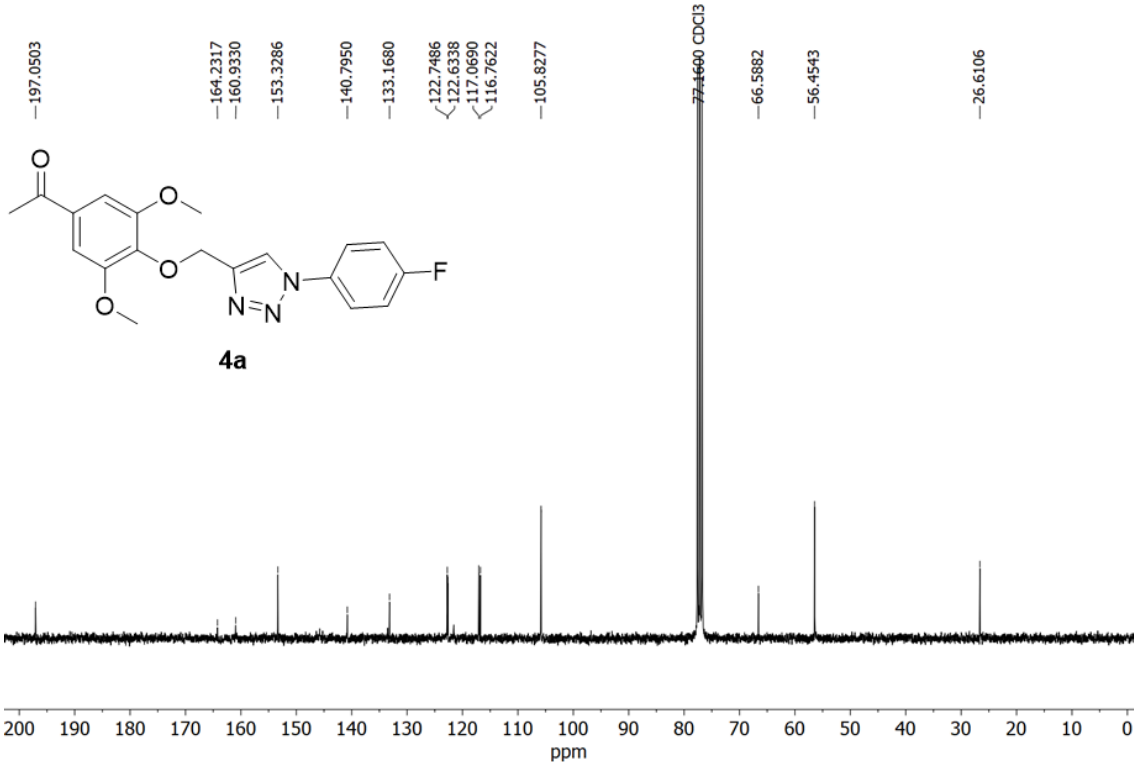

Figure S5 - ^{1}H NMR and ^{13}C NMR spectra of compound **4a**.

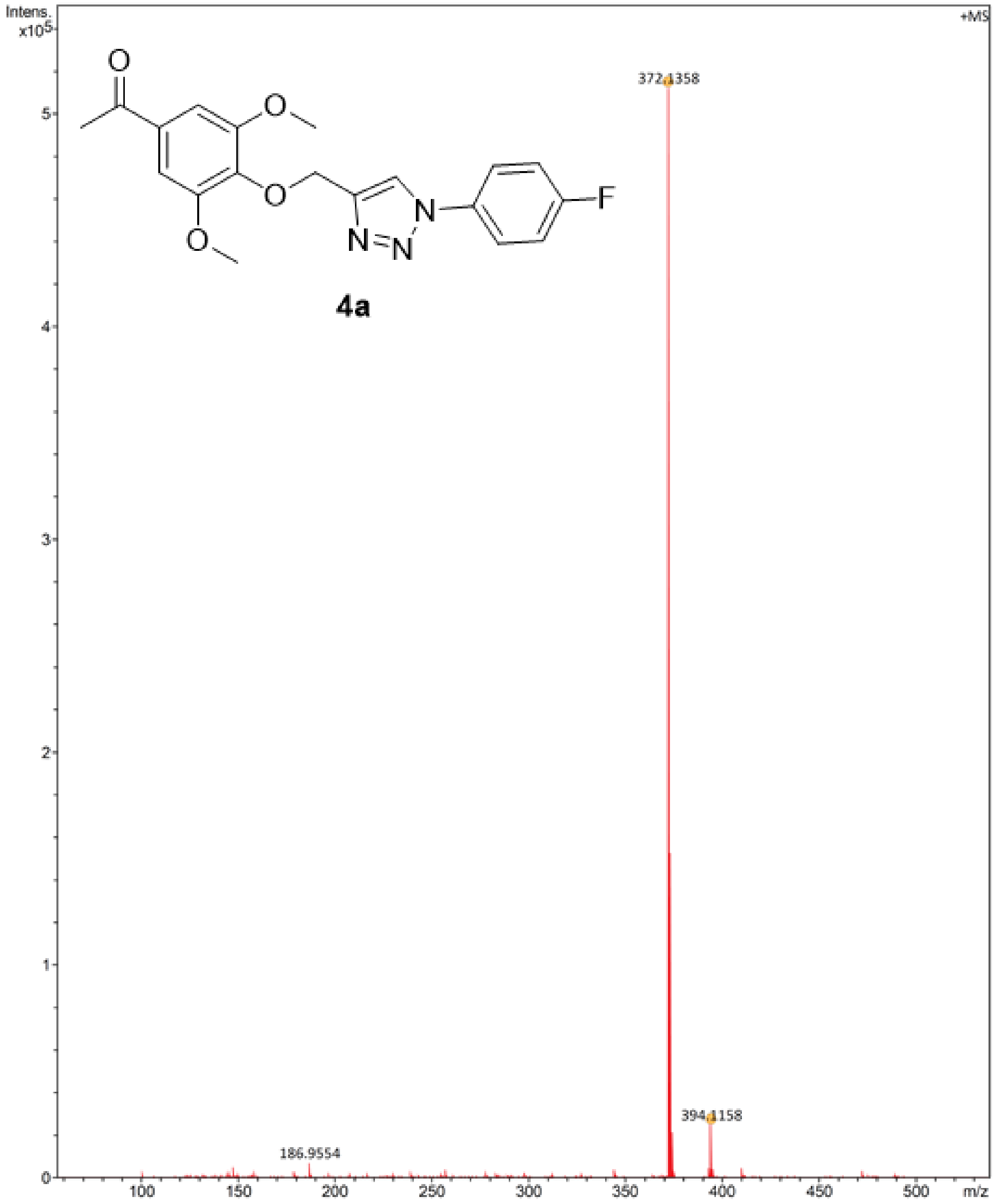

Meas. m/z	Formula	m/z	err [ppm]
394.1158	$C_{19}H_{18}FN_3NaO_4$	394.1174	3.9

Figure S6 - HRMS spectrum of compound **4a**.

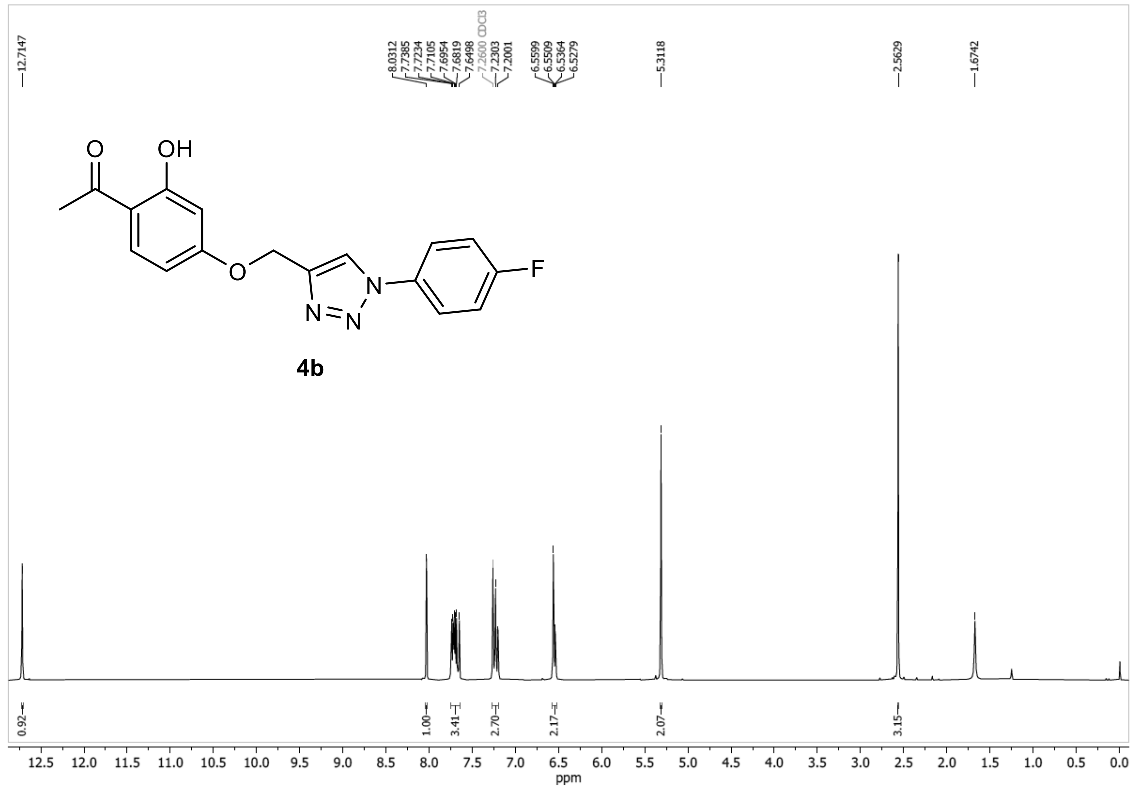

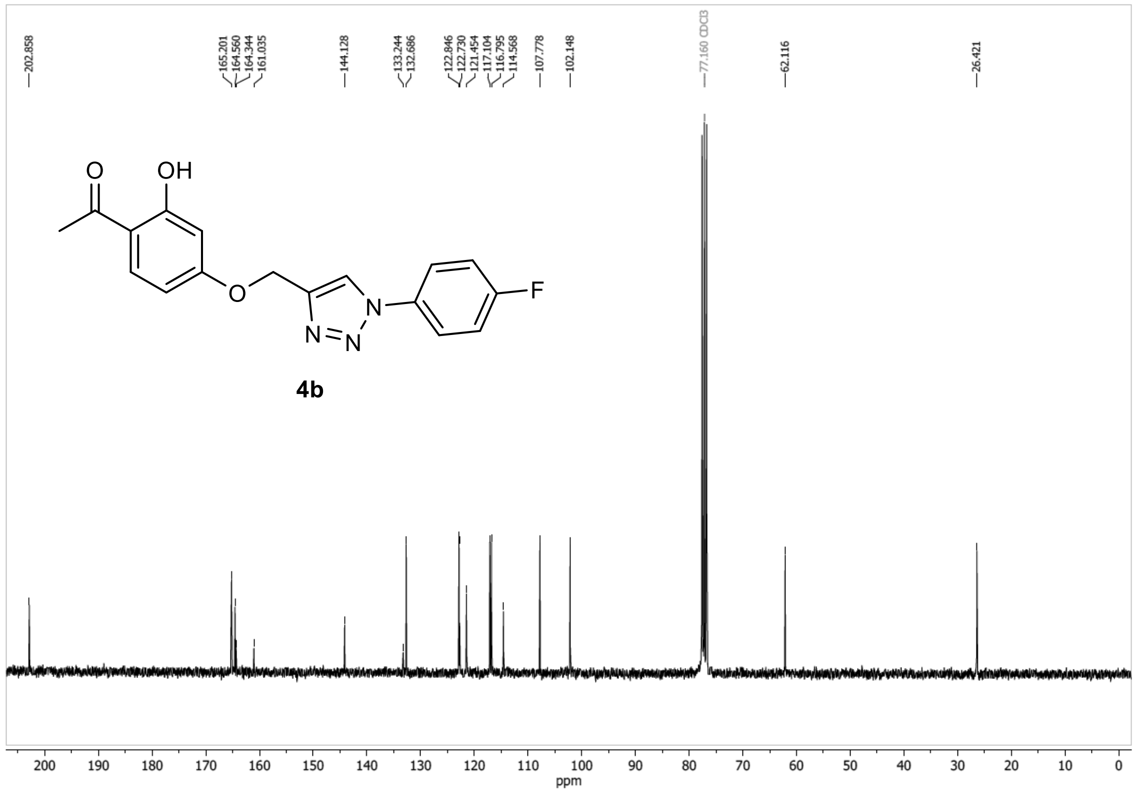

Figure S7 - ^{1}H NMR and ^{13}C NMR spectra of compound **4b**.

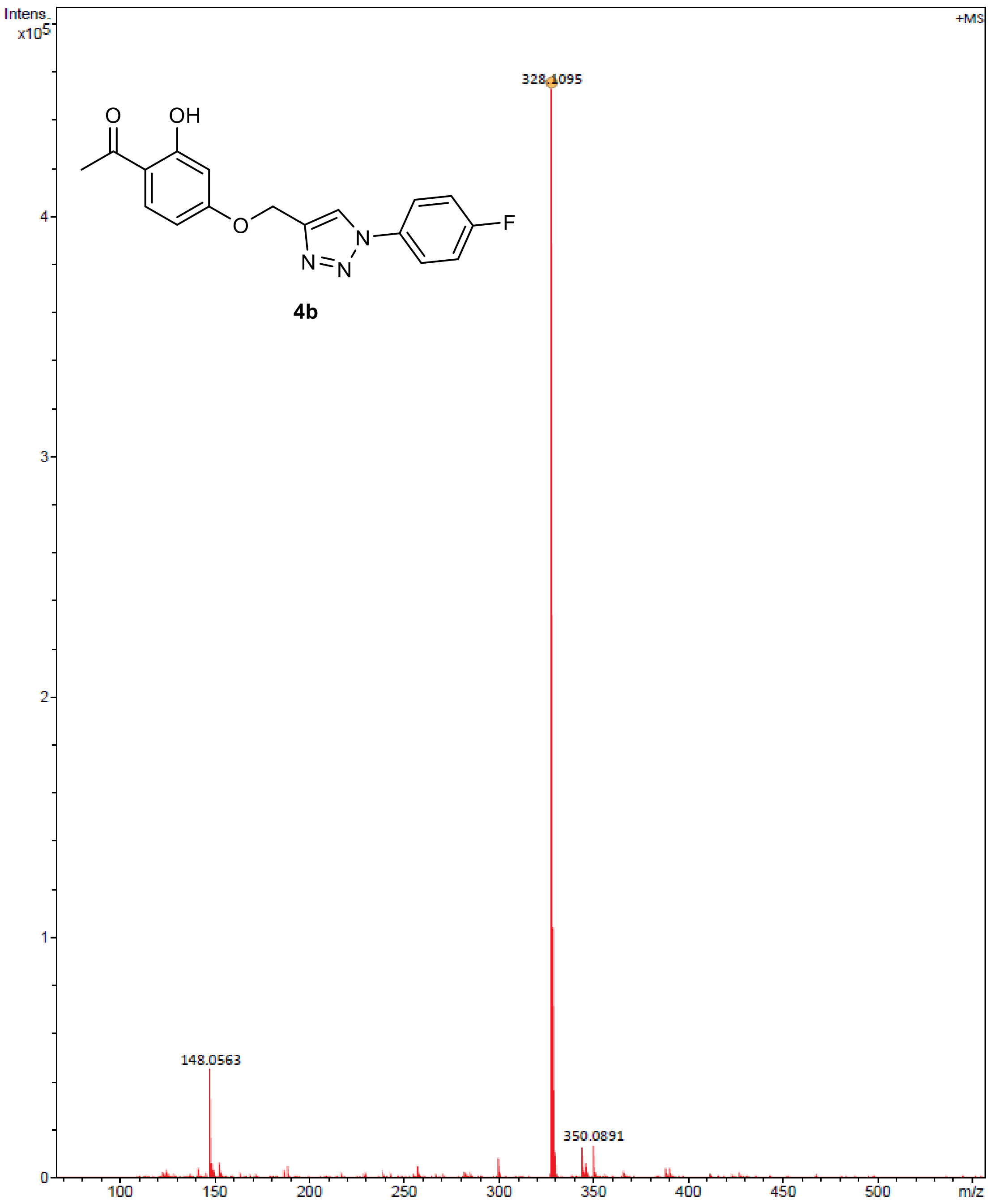

Mass Spectrum Molecular Formula Report

Meas. m/z	#	Ion Formula	m/z	err [ppm]	mSigma	# mSigma	Score	rdb	e¯ Conf	N-Rule
328.1095	1	C17H15FN3O3	328.1092	-1.1	18.6	1	100.00	11.5	even	ok

Figure S8 - HRMS spectrum of compound **4b**.

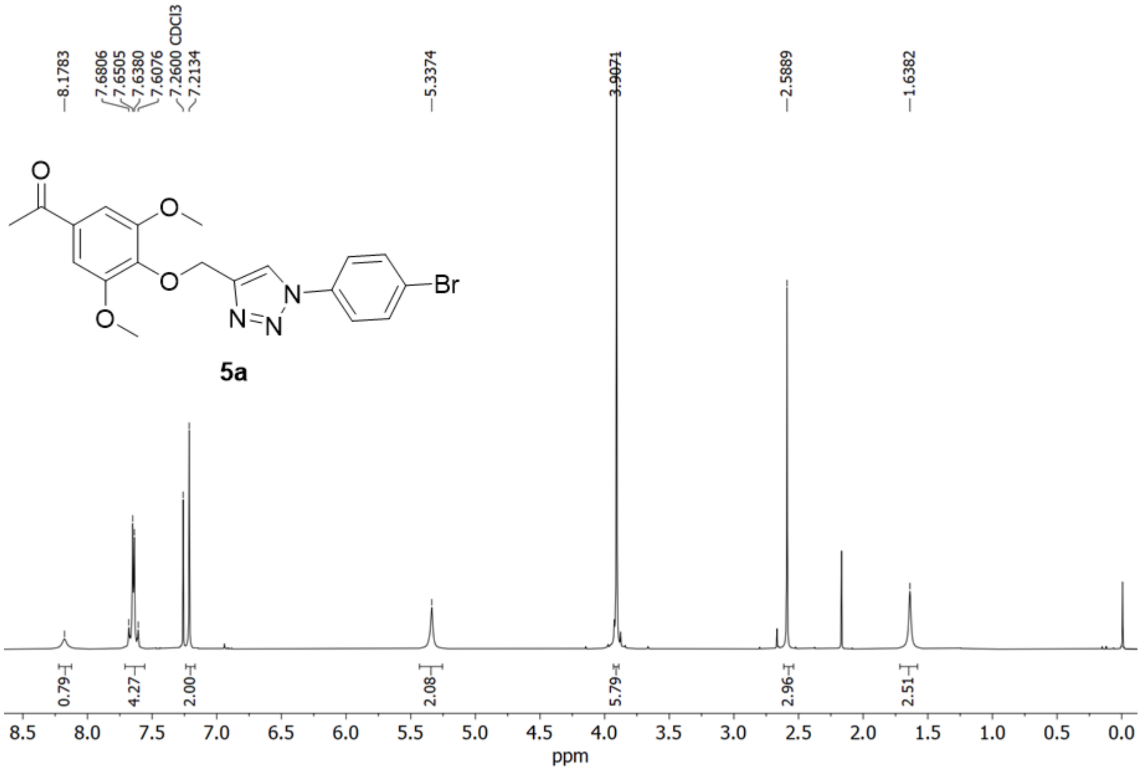

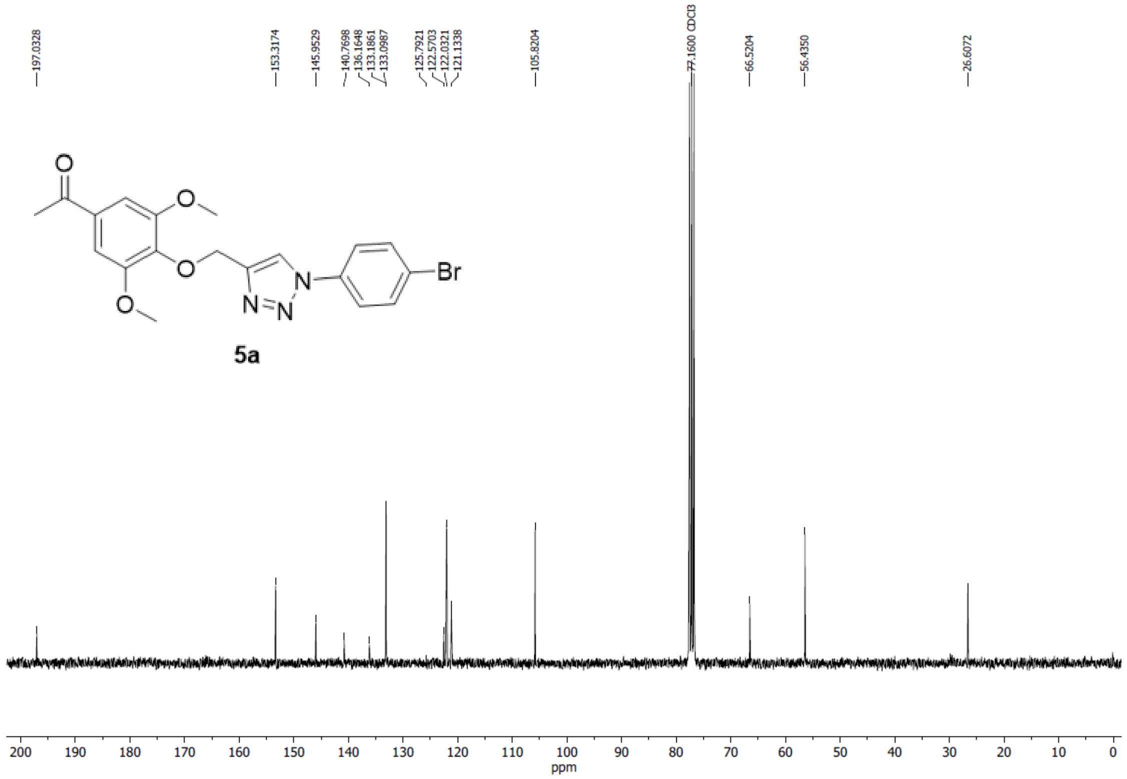

Figure S9 - ^{1}H NMR and ^{13}C NMR spectra of compound **5a**.

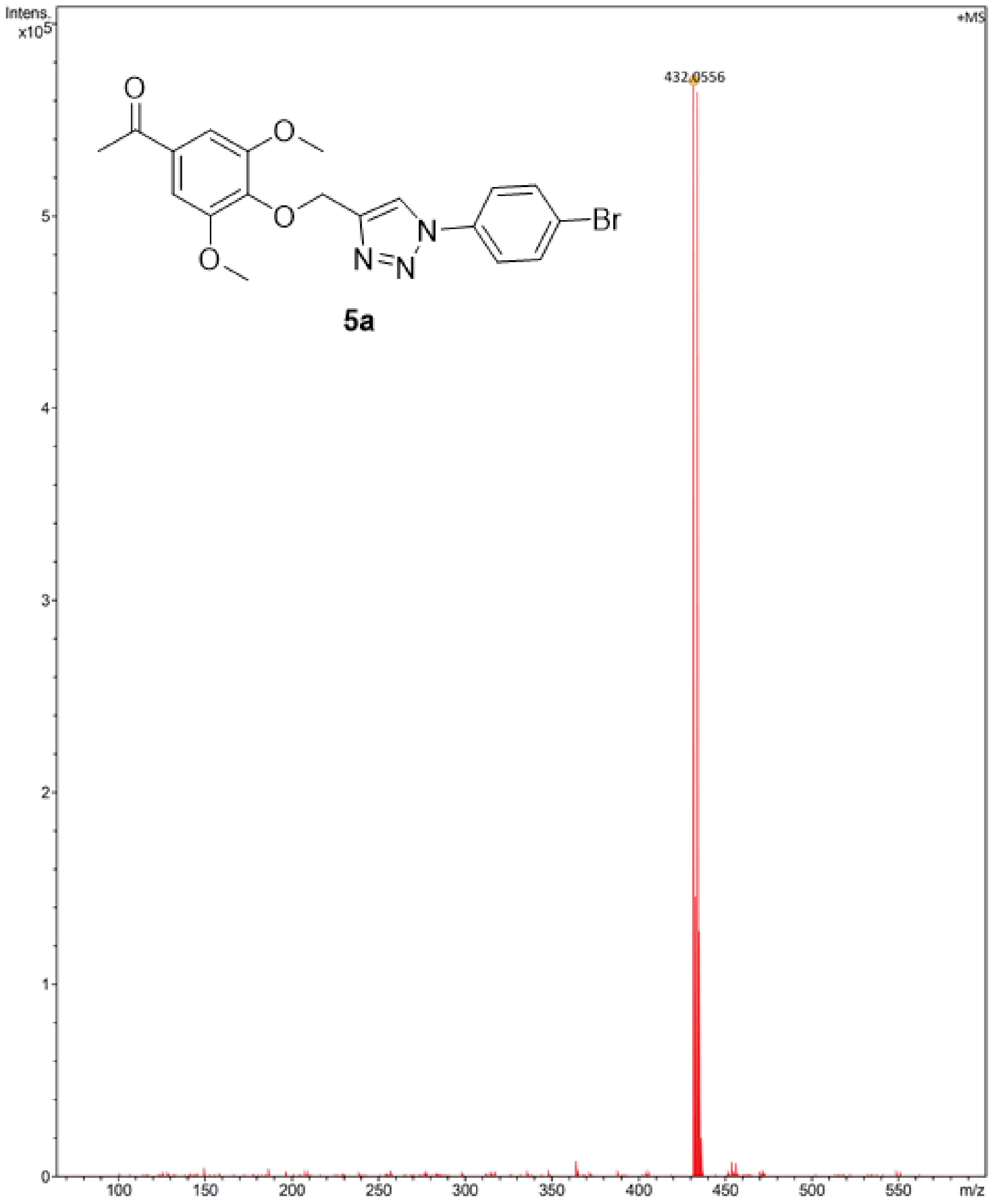

Meas. m/z	Formula	m/z	err [ppm]
432.0556	$C_{19}H_{19}BrN_3O_4$	432.0553	-0.6

Figure S10 - HRMS spectrum of compound **5a**.

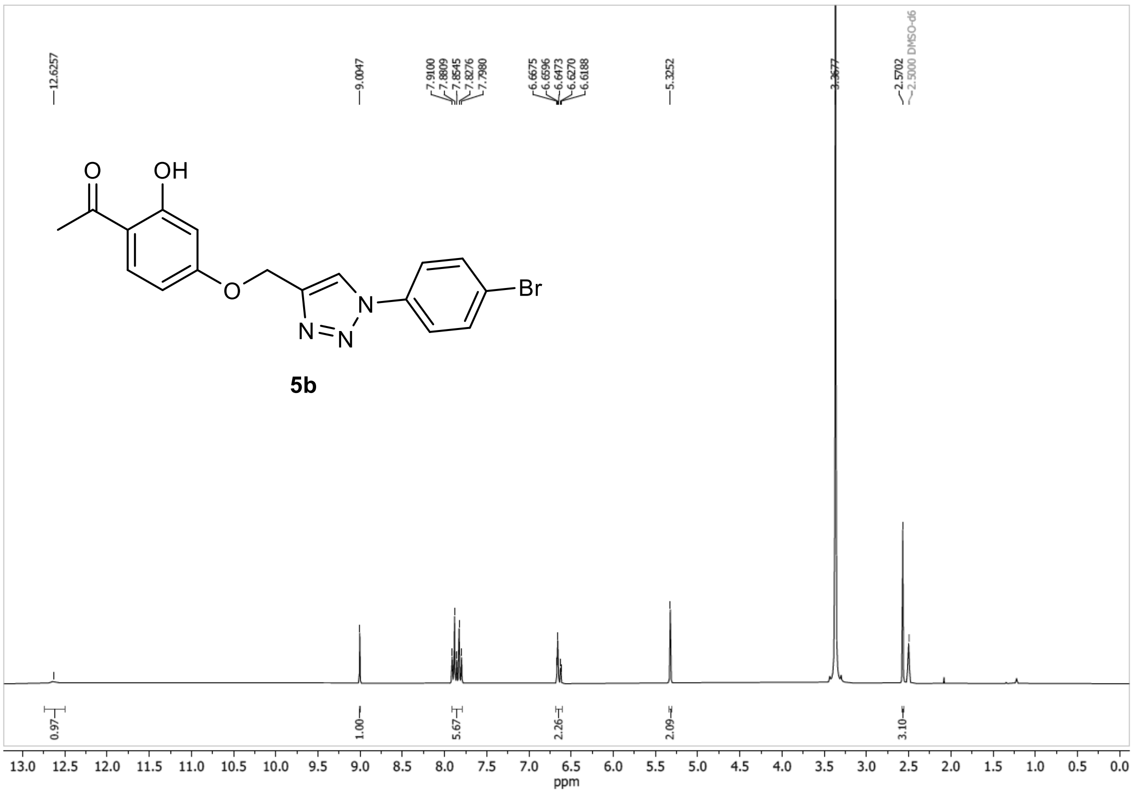

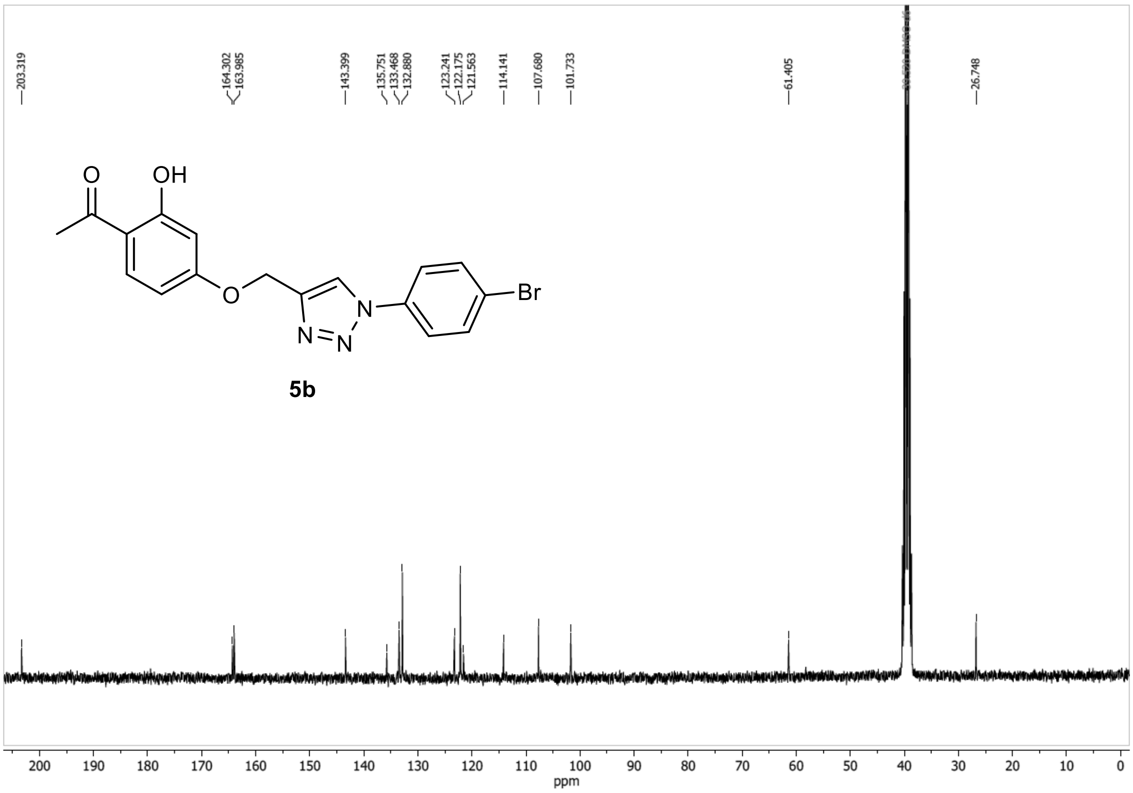

Figure S11 - ^{1}H NMR and ^{13}C NMR spectra of compound **5b**.

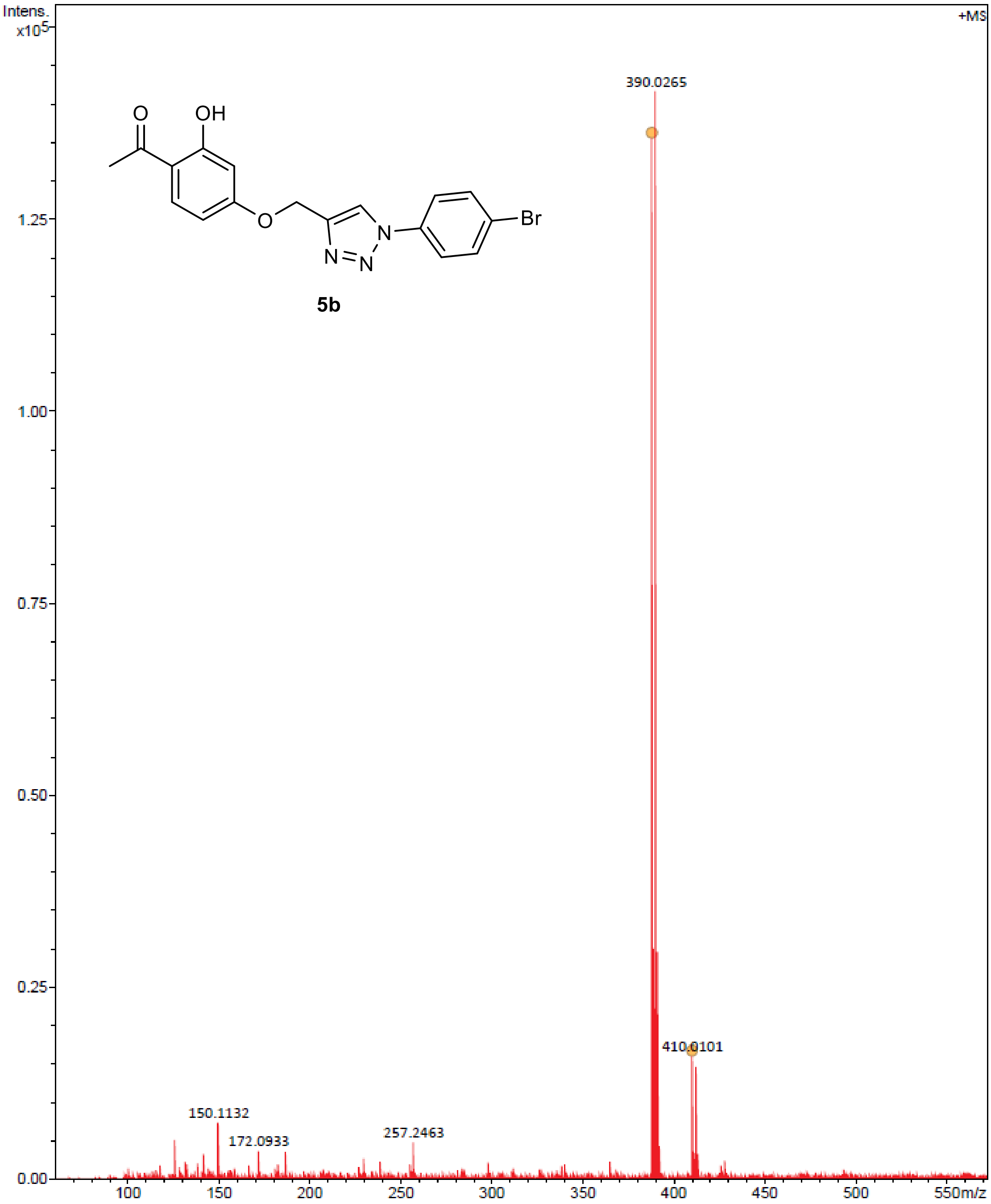

Mass Spectrum Molecular Formula Report

Meas. m/z	#	Ion Formula	m/z	err [ppm]	mSigma	# mSigma	Score	rdb	e⁻ Conf	N-Rule
388.0292	1	C17H15BrN3O3	388.0291	-0.1	21.7	2	100.00	11.5	even	ok

Figure S12 - HRMS spectrum of compound **5b**.

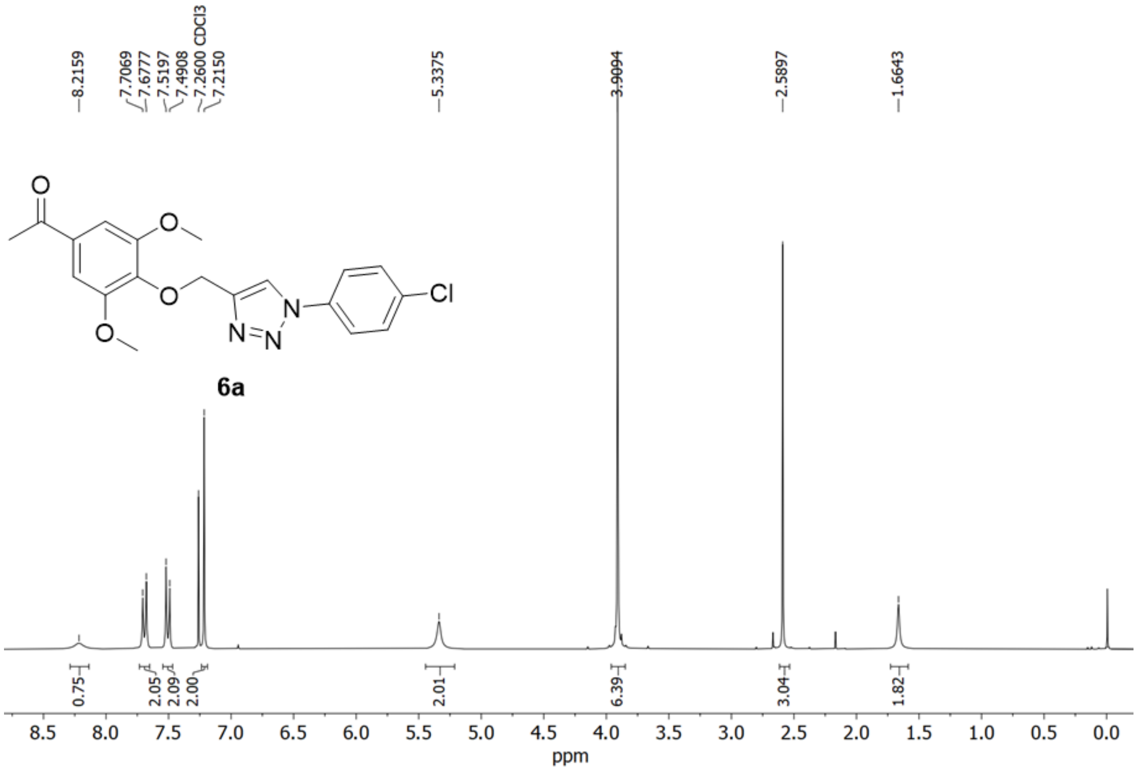

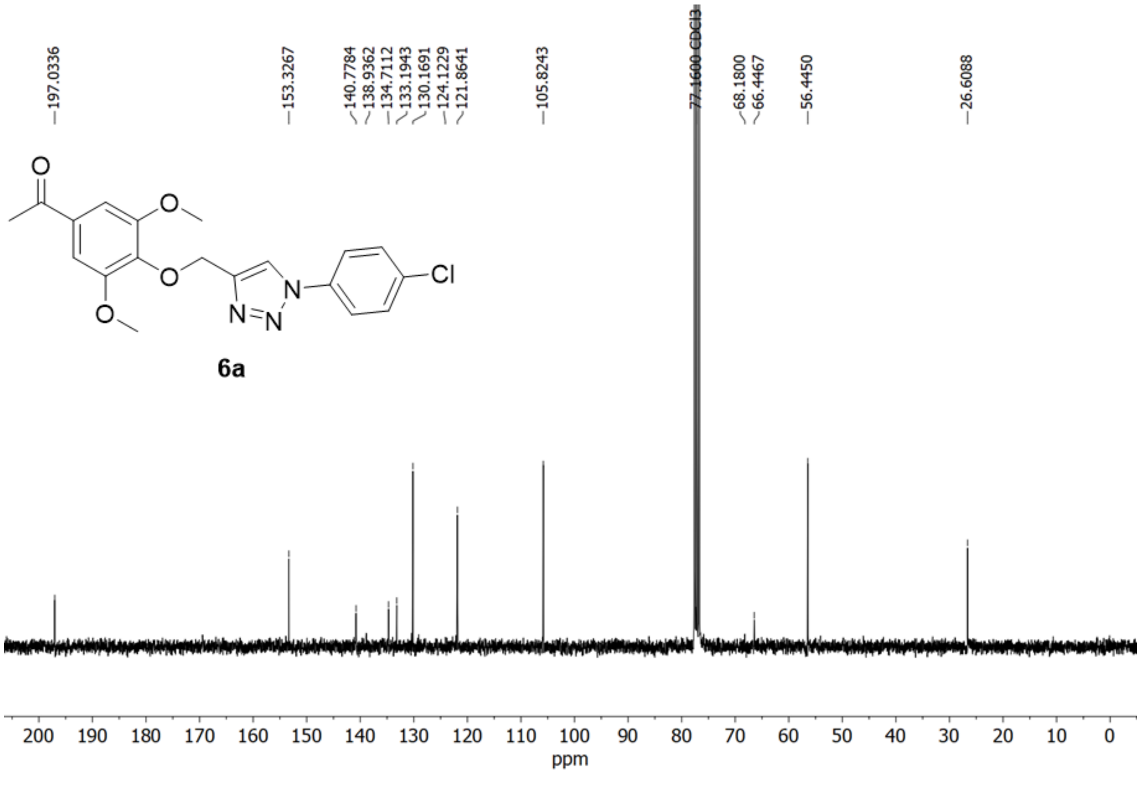

Figure S13 - ^{1}H NMR and ^{13}C NMR spectra of compound **6a**.

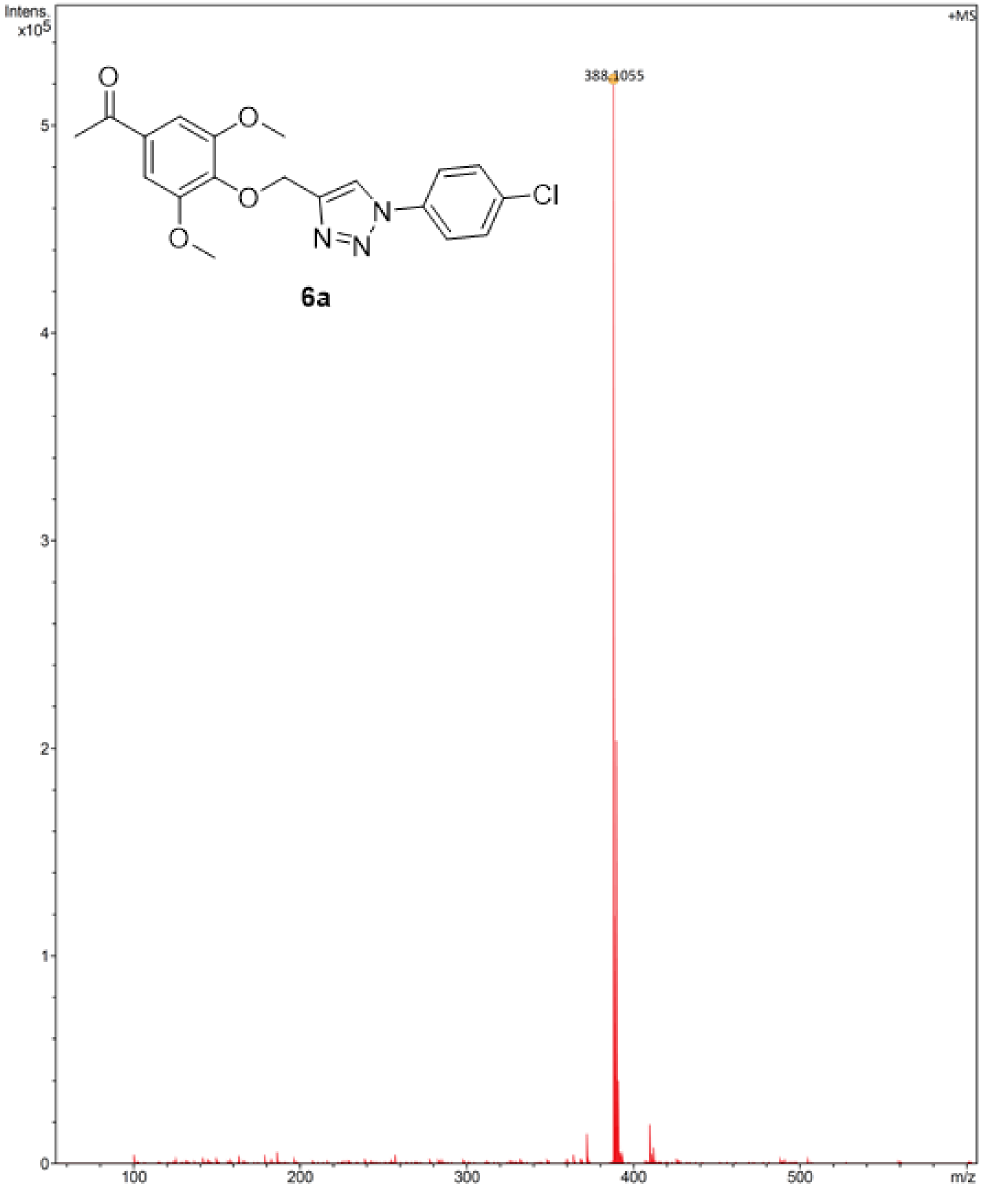

Meas. m/z	Formula	m/z	err [ppm]
388.1055	$C_{19}H_{19}ClN_3O_4$	388.1059	1.0

Figure S14 - HRMS spectrum of compound **6a**.

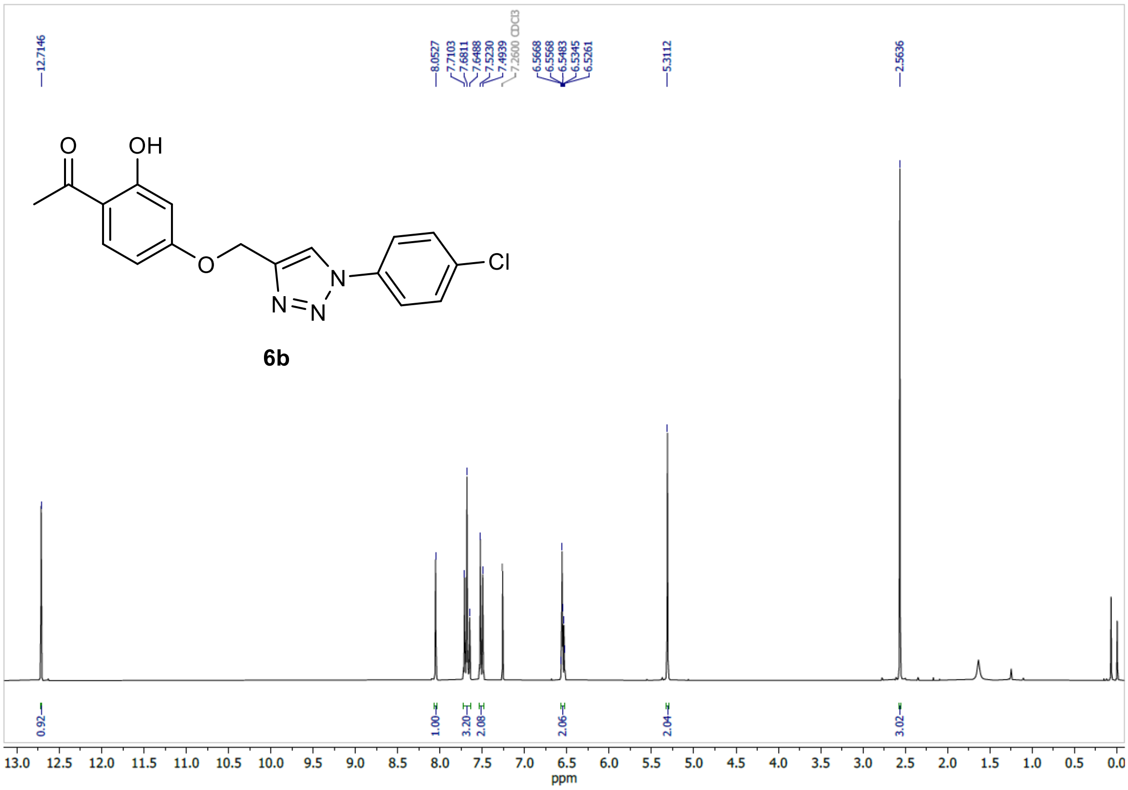

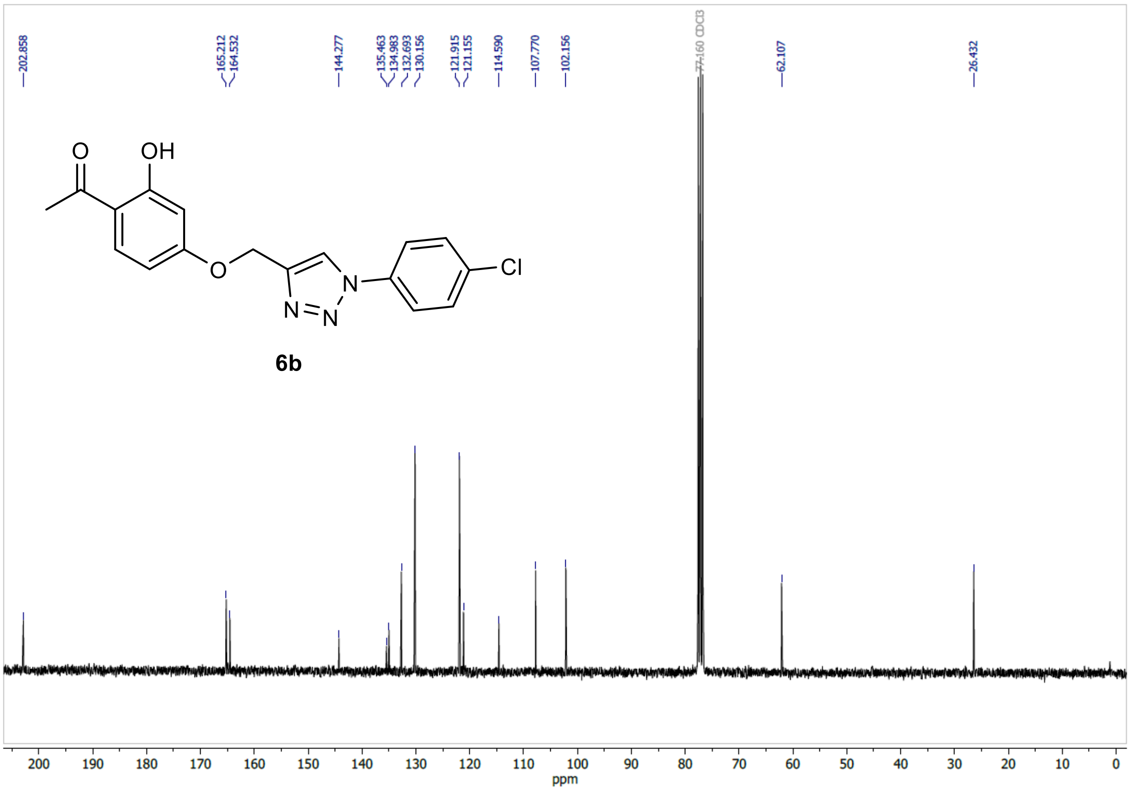

Figure S15 - ^{1}H NMR and ^{13}C NMR spectra of compound **6b**.

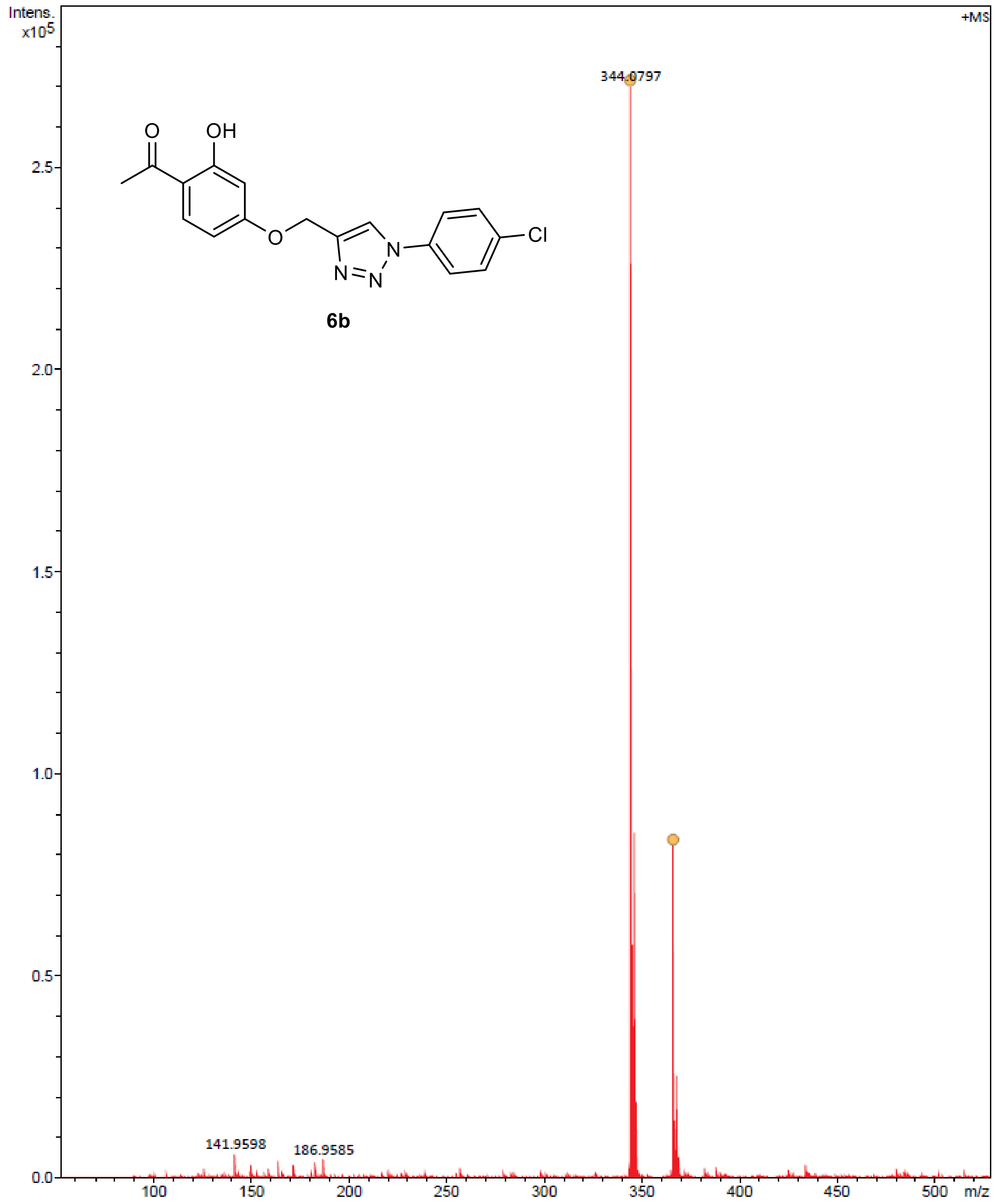

Mass Spectrum Molecular Formula Report

Meas. m/z	#	Ion Formula	m/z	err [ppm]	mSigma	# mSigma	Score	rdb	e⁻ Conf	N-Rule
344.0797	1	C17H15ClN3O3	344.0796	-0.2	16.8	1	100.00	11.5	even	ok

Figure S16 - HRMS spectrum of compound **6b**.

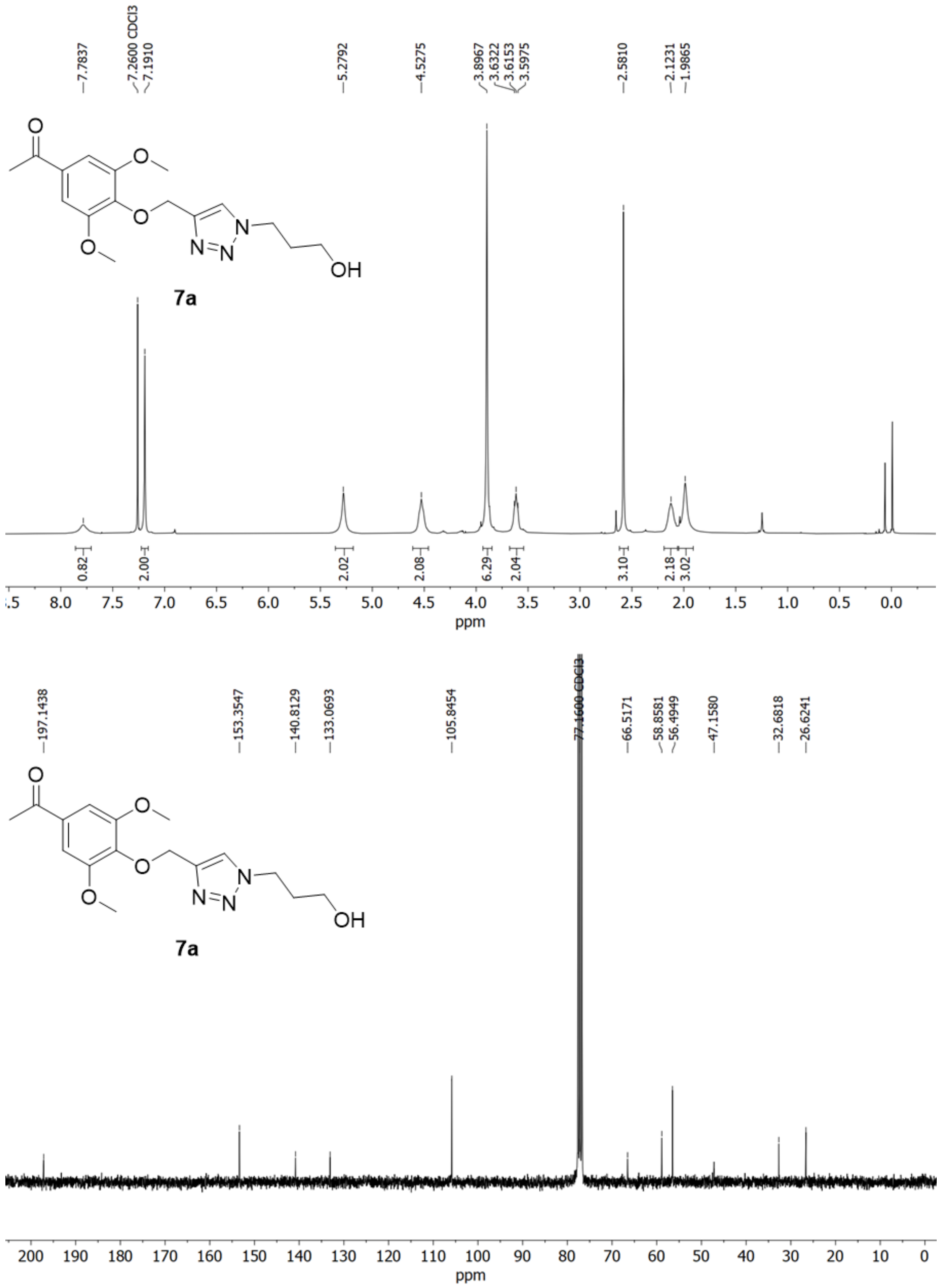

Figure S17 - ^{1}H NMR and ^{13}C NMR spectra of compound **7a**.

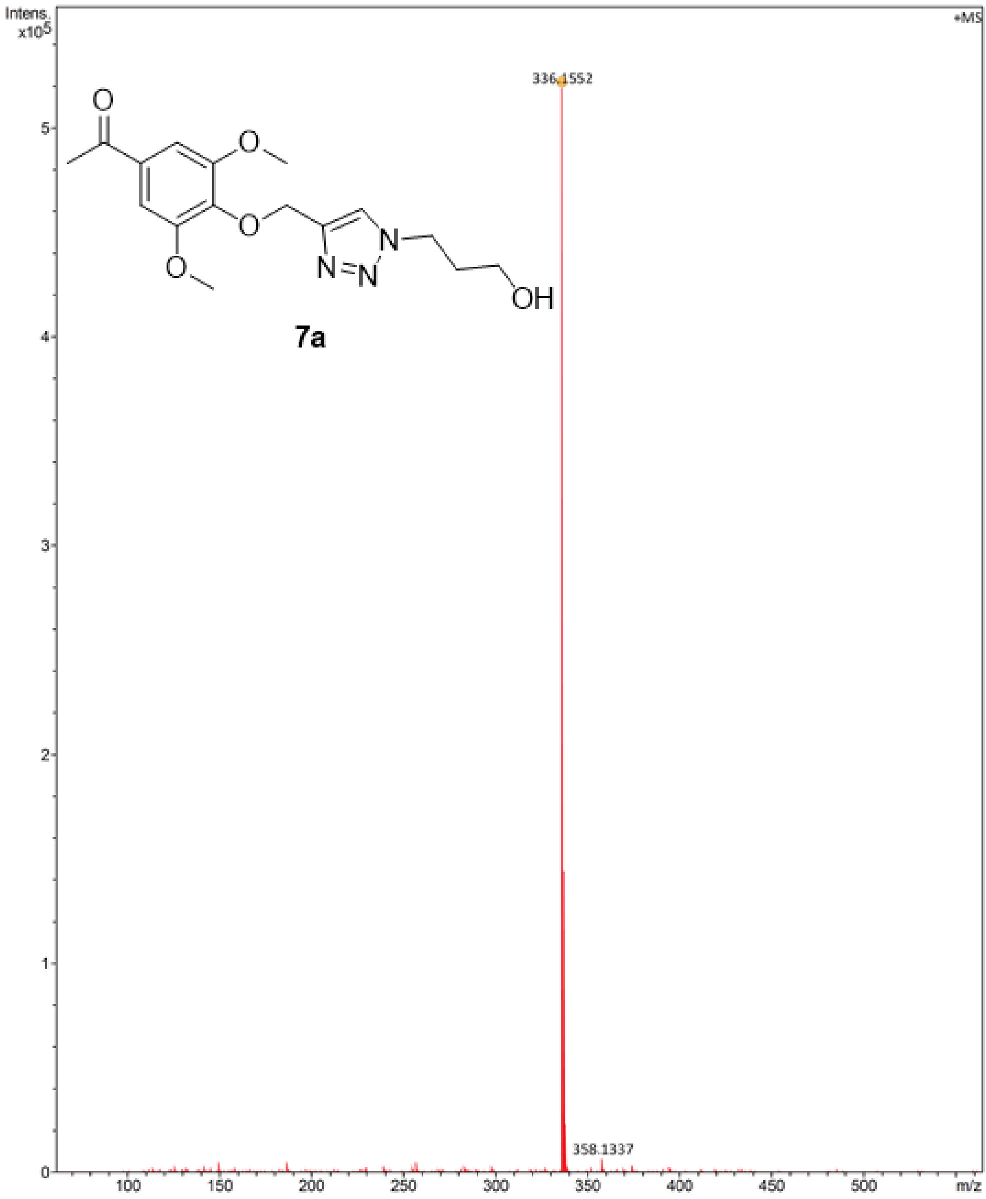

Meas. m/z	Formula	m/z	err [ppm]
336.1552	$C_{16}H_{22}N_3O_5$	336.1554	0.5

Figure S18 - HRMS spectrum of compound **7a**.

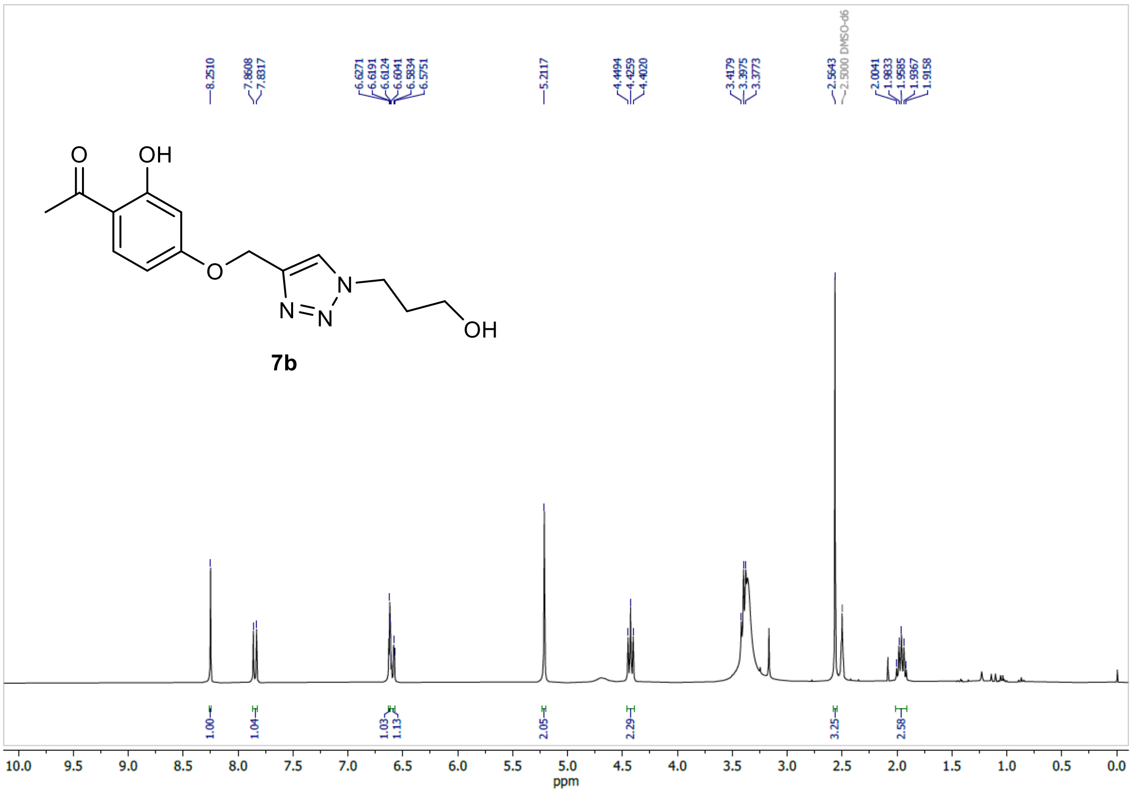

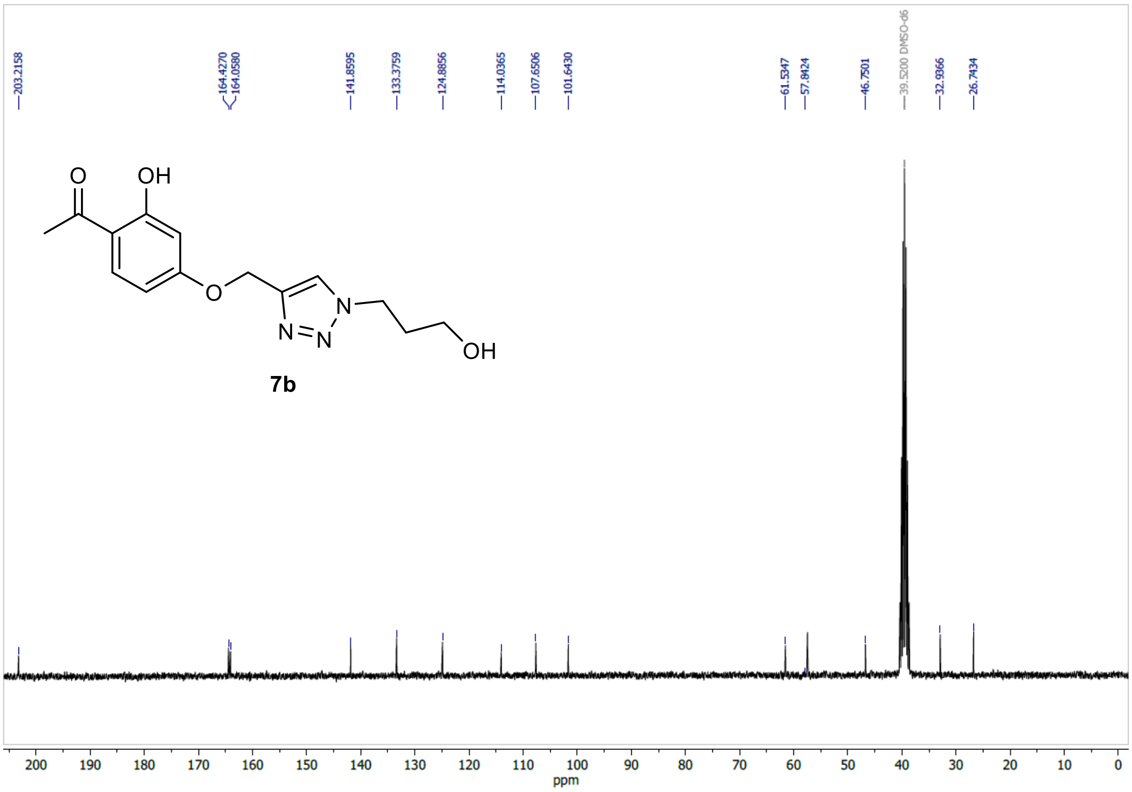

Figure S19 - ^{1}H NMR and ^{13}C NMR spectra of compound **7b**.

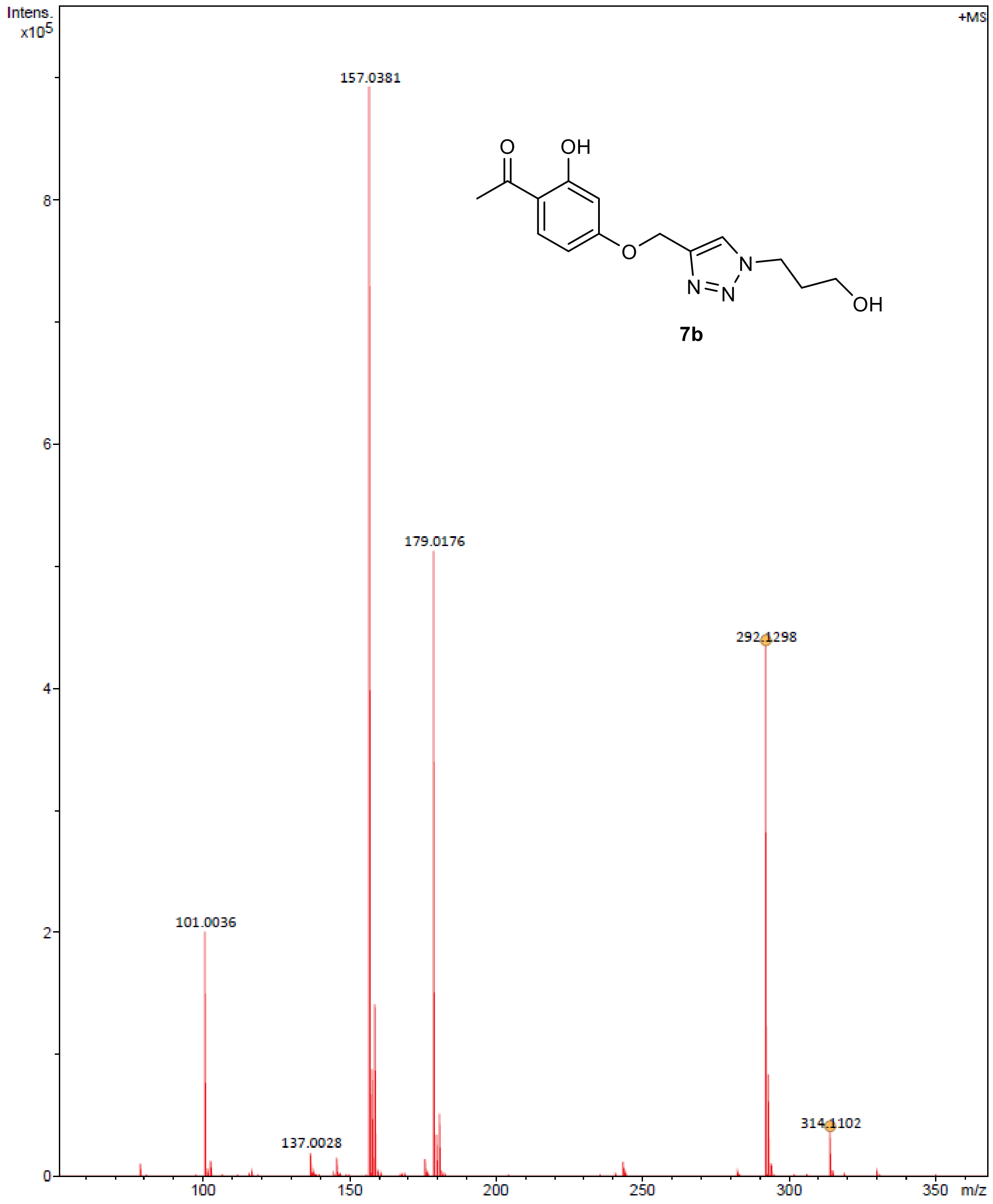

Mass Spectrum Molecular Formula Report

Meas. m/z	#	Ion Formula	m/z	err [ppm]	mSigma	# mSigma	Score	rdb	e⁻ Conf	N-Rule
292.1298	1	C14H18N3O4	292.1292	-2.0	15.6	3	77.22	7.5	even	ok

Figure S20 - HRMS spectrum of compound **7b**.

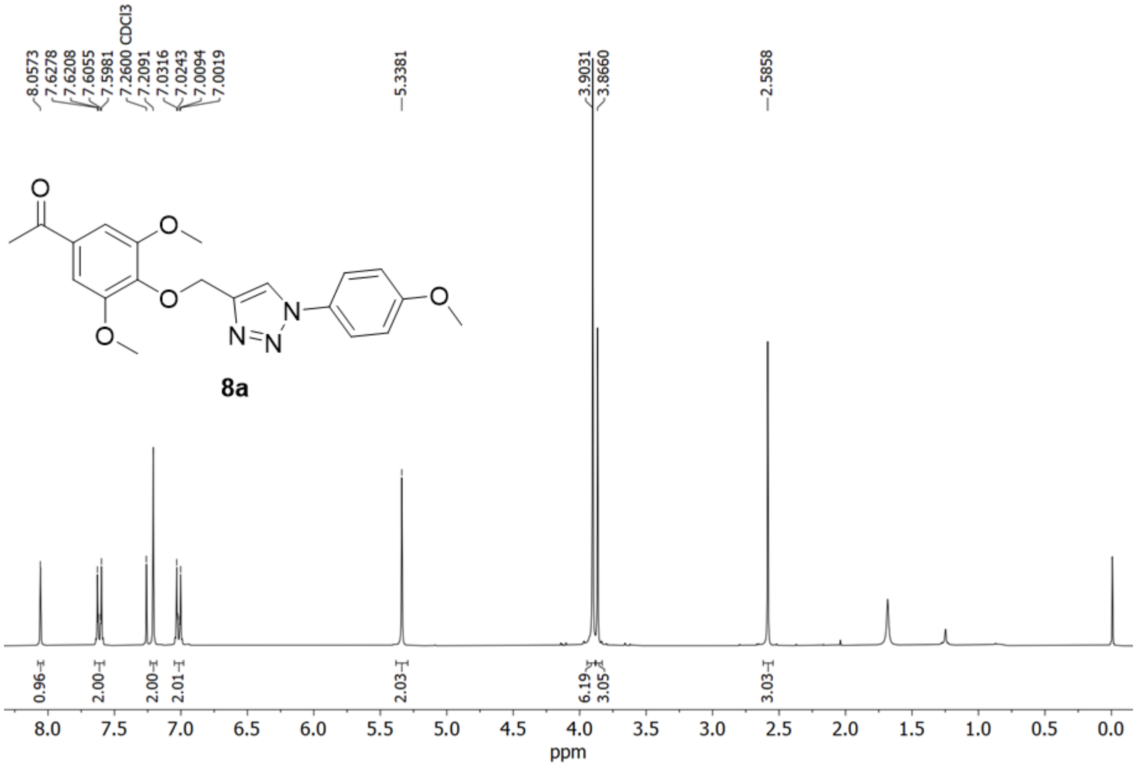

8a

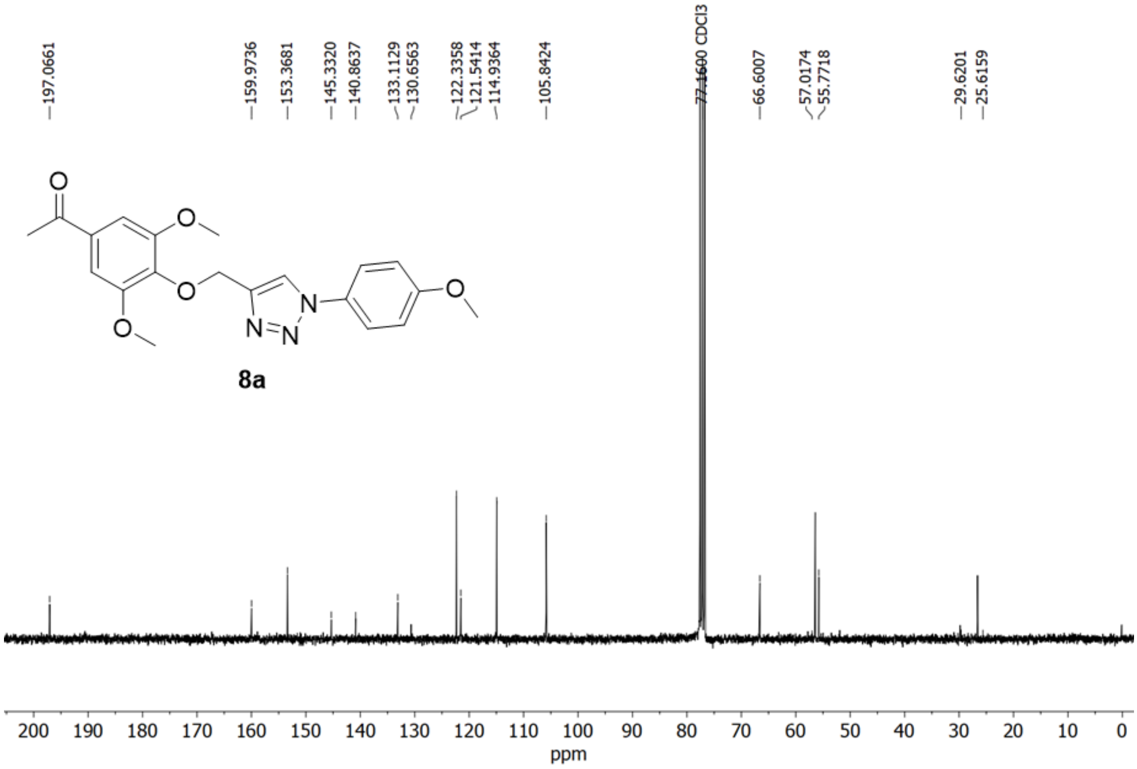

8a

Figure S21 - ^{1}H NMR and ^{13}C NMR spectra of compound **8a**.

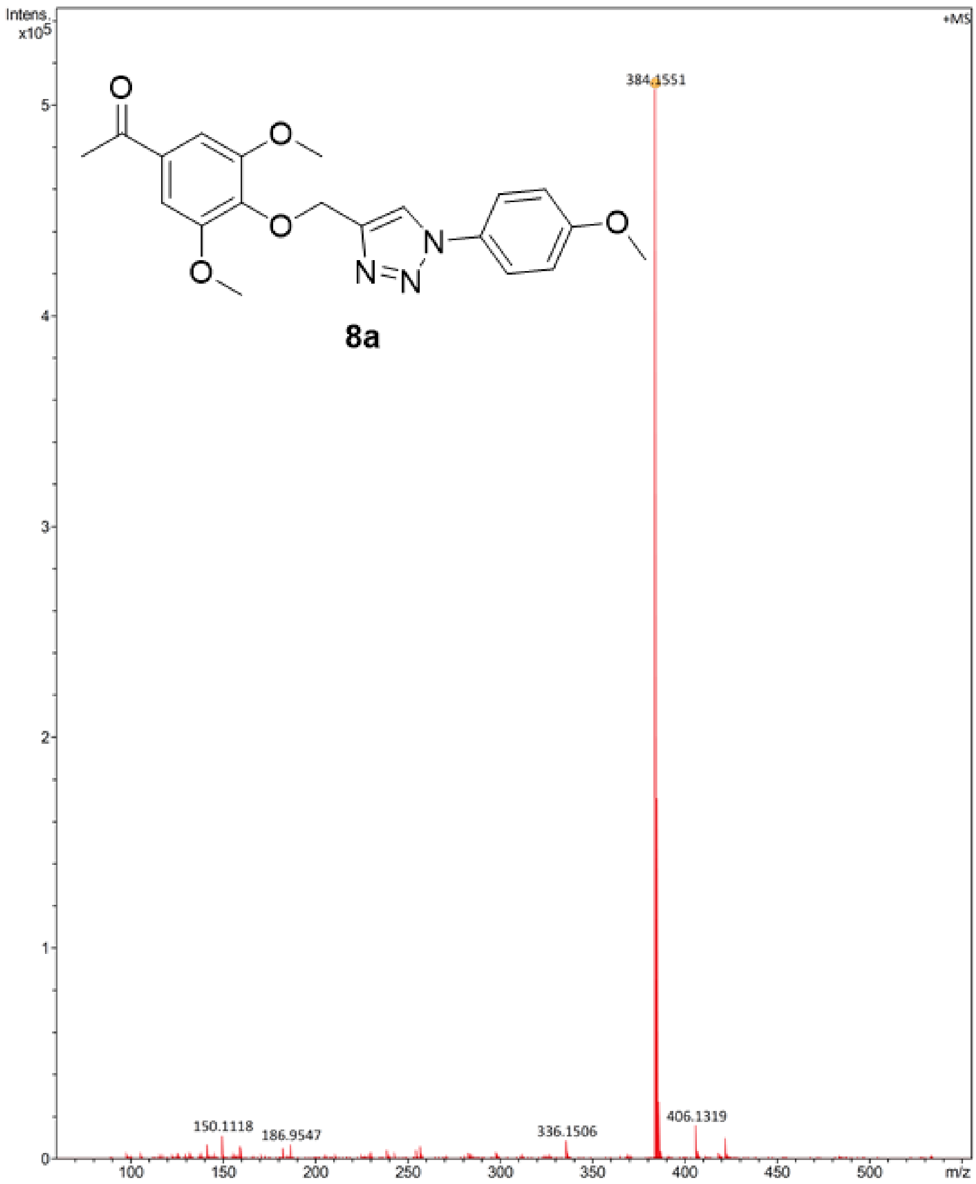

Meas. m/z	Formula	m/z	err [ppm]
384.1551	$C_{20}H_{22}N_3O_5$	384.1554	0.7

Figure S22 - HRMS spectrum of compound **8a**.

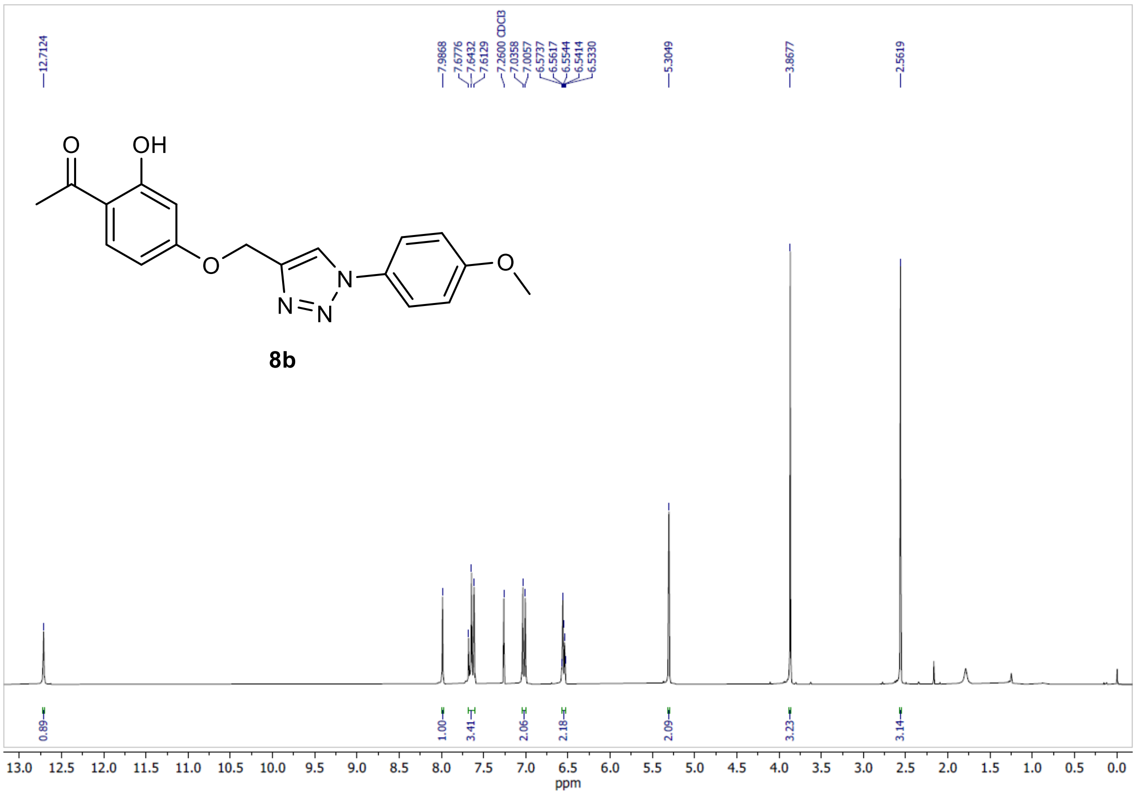

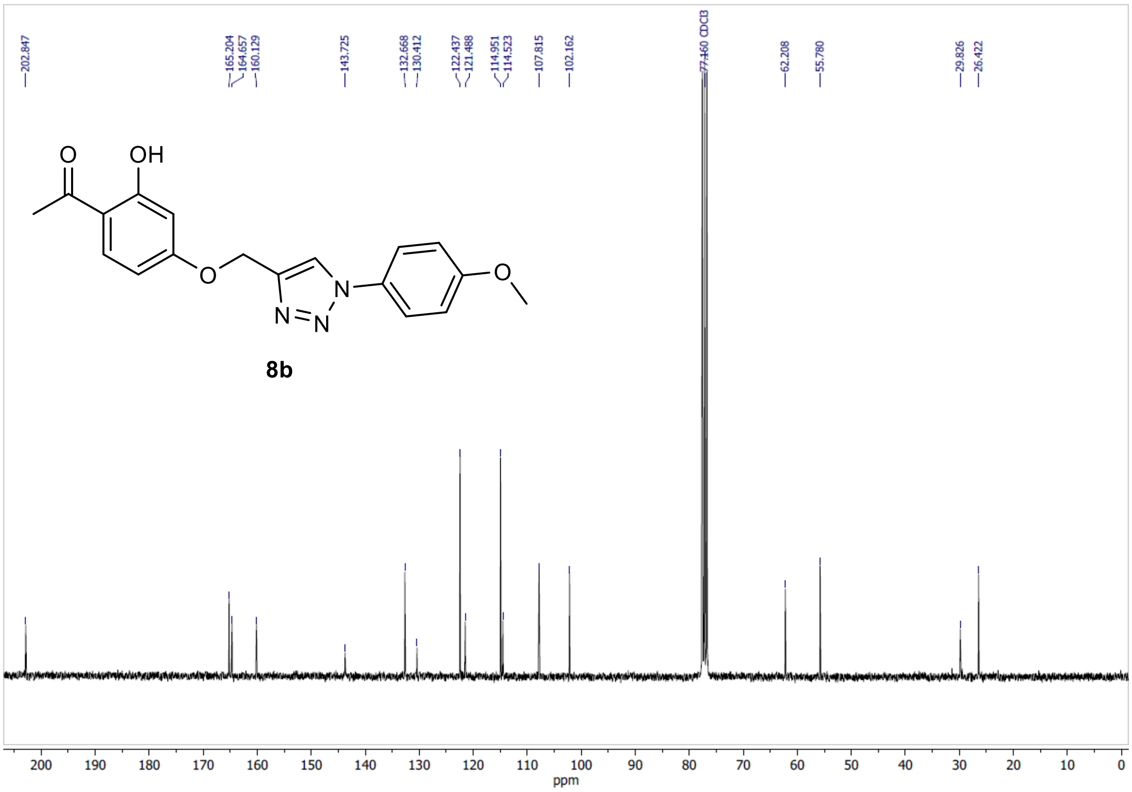

Figure S23 - ^{1}H NMR and ^{13}C NMR spectra of compound **8b**.

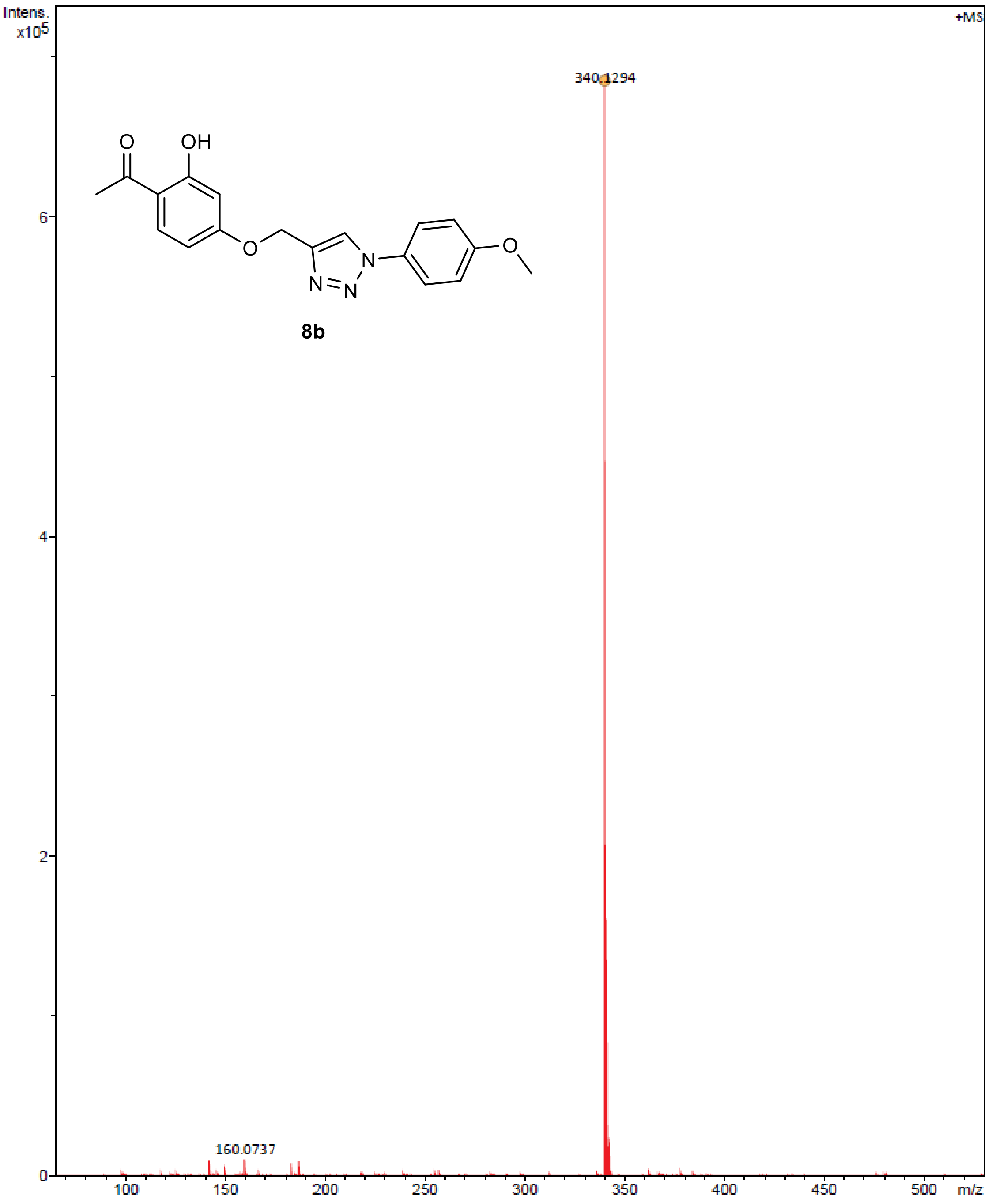

Mass Spectrum Molecular Formula Report

Meas. m/z	#	Ion Formula	m/z	err [ppm]	mSigma	# mSigma	Score	rdb	e⁻ Conf	N-Rule
340.1294	1	C18H18N3O4	340.1292	-0.6	15.7	1	100.00	11.5	even	ok

Figure S24 - HRMS spectrum of compound **8b**.

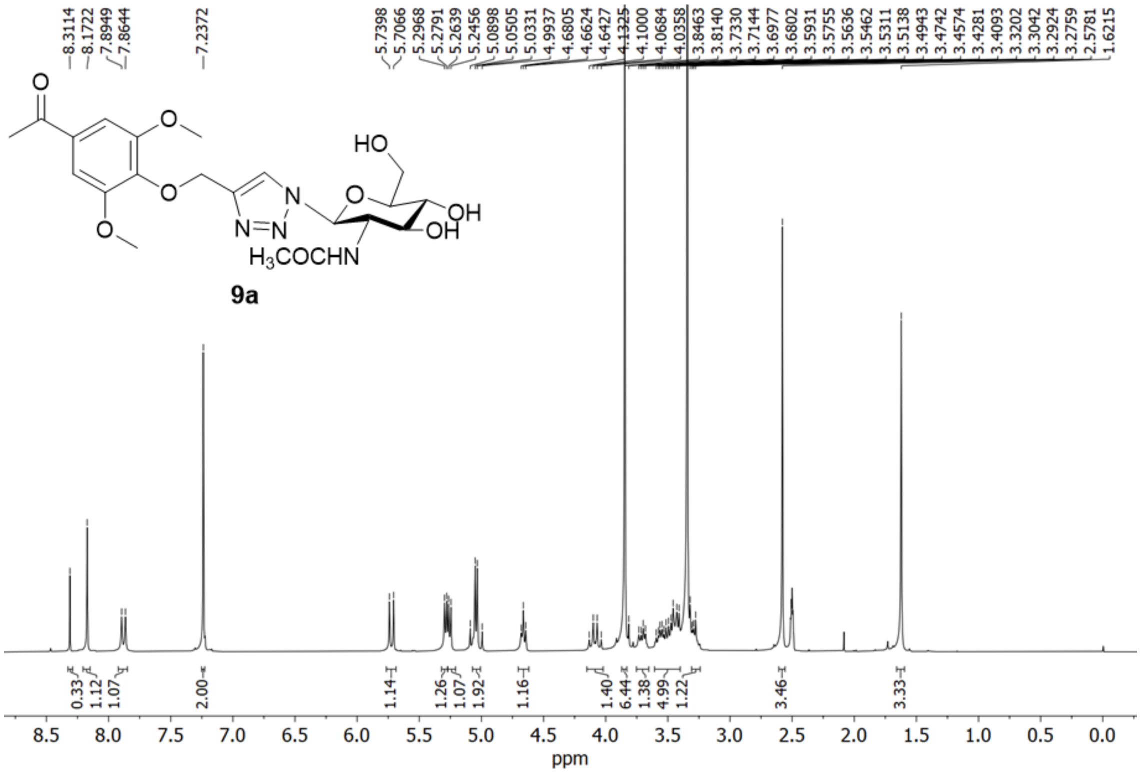

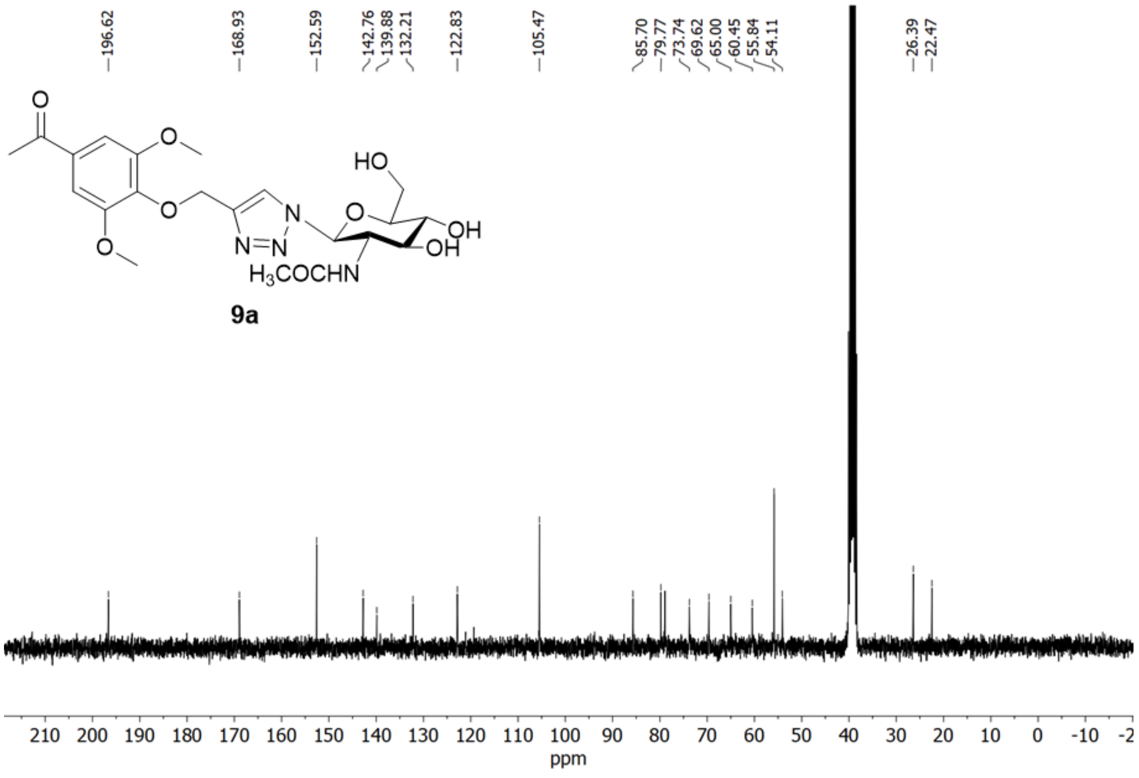

Figure S25 - ^{1}H NMR and ^{13}C NMR spectra of compound **9a**.

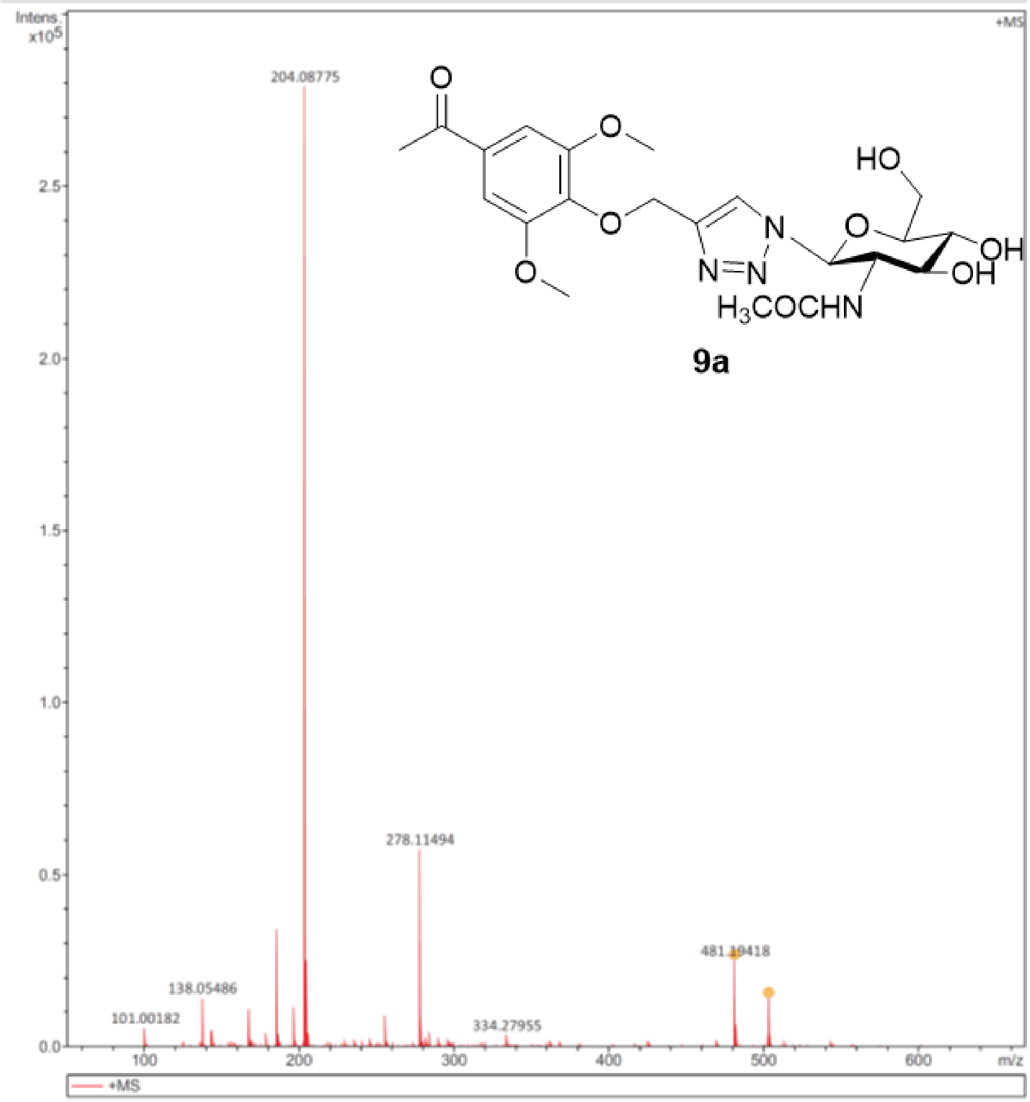

Meas. m/z	Formula	m/z	err [ppm]
503.17612	$C_{21}H_{28}N_4O_4Na$	503.17485	-2.53

Figure S26 - HRMS spectrum of compound **9a**.

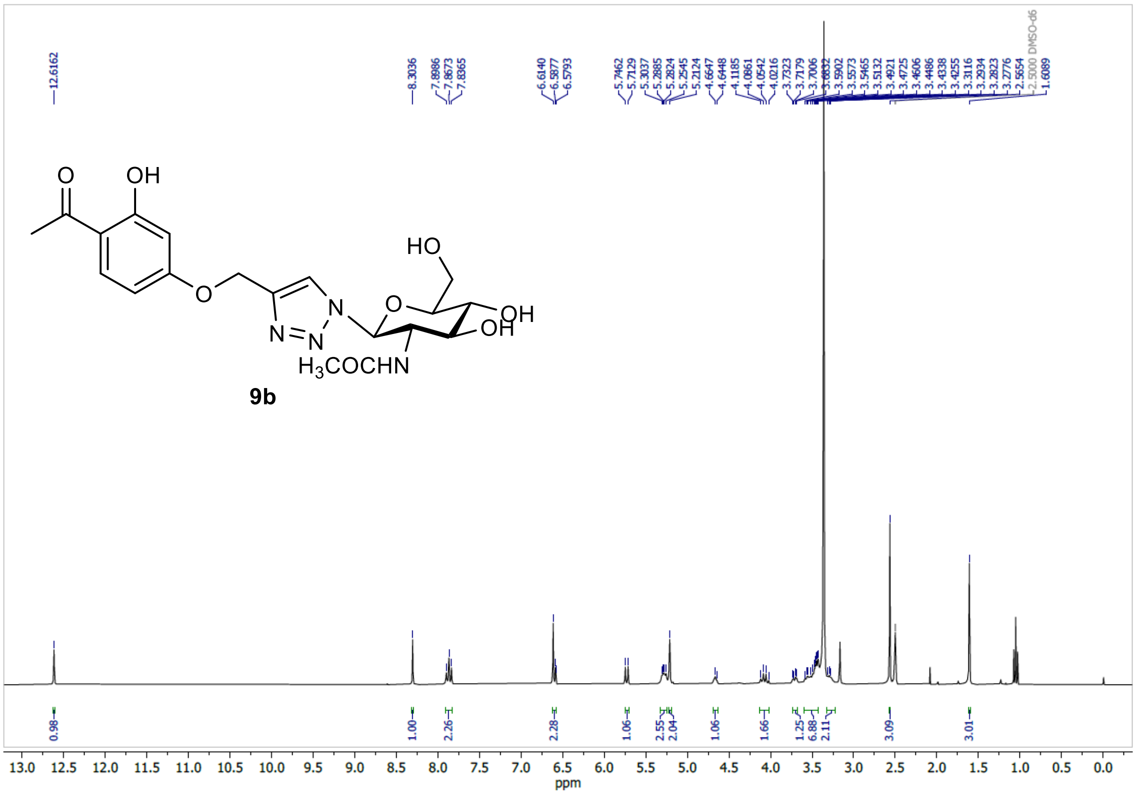

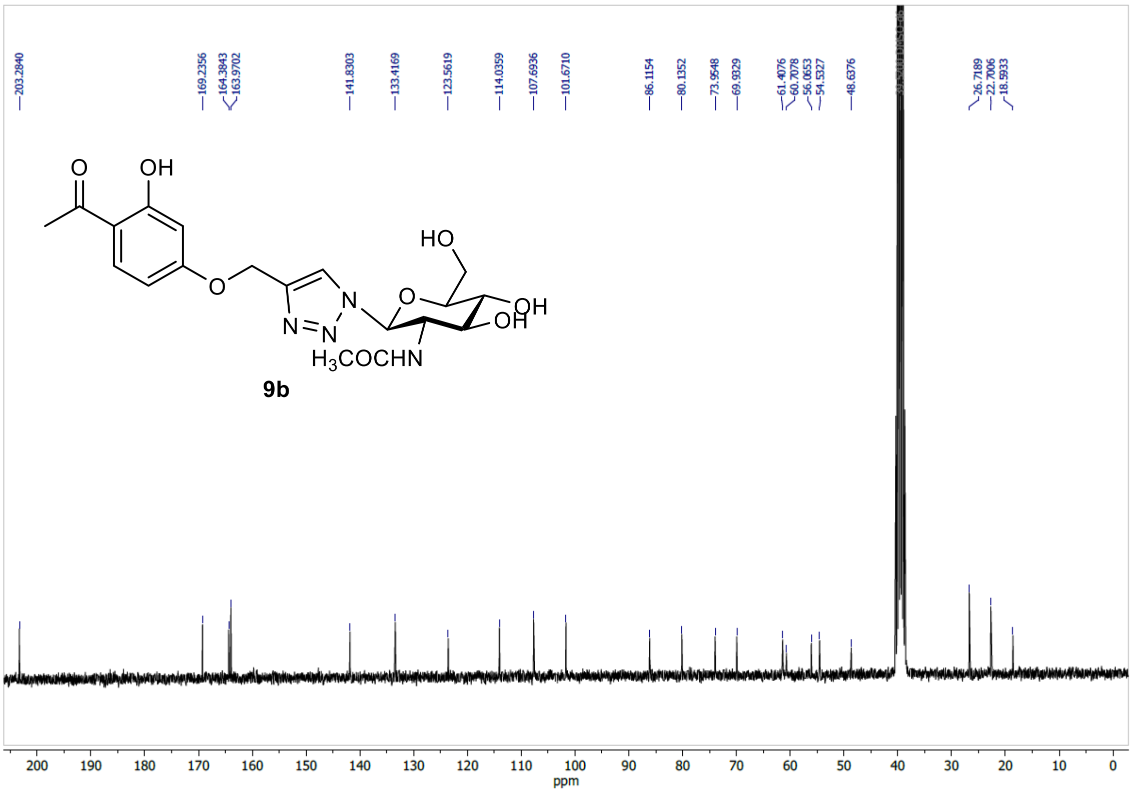

Figure S27 - ^{1}H NMR and ^{13}C NMR spectra of compound **9b**.

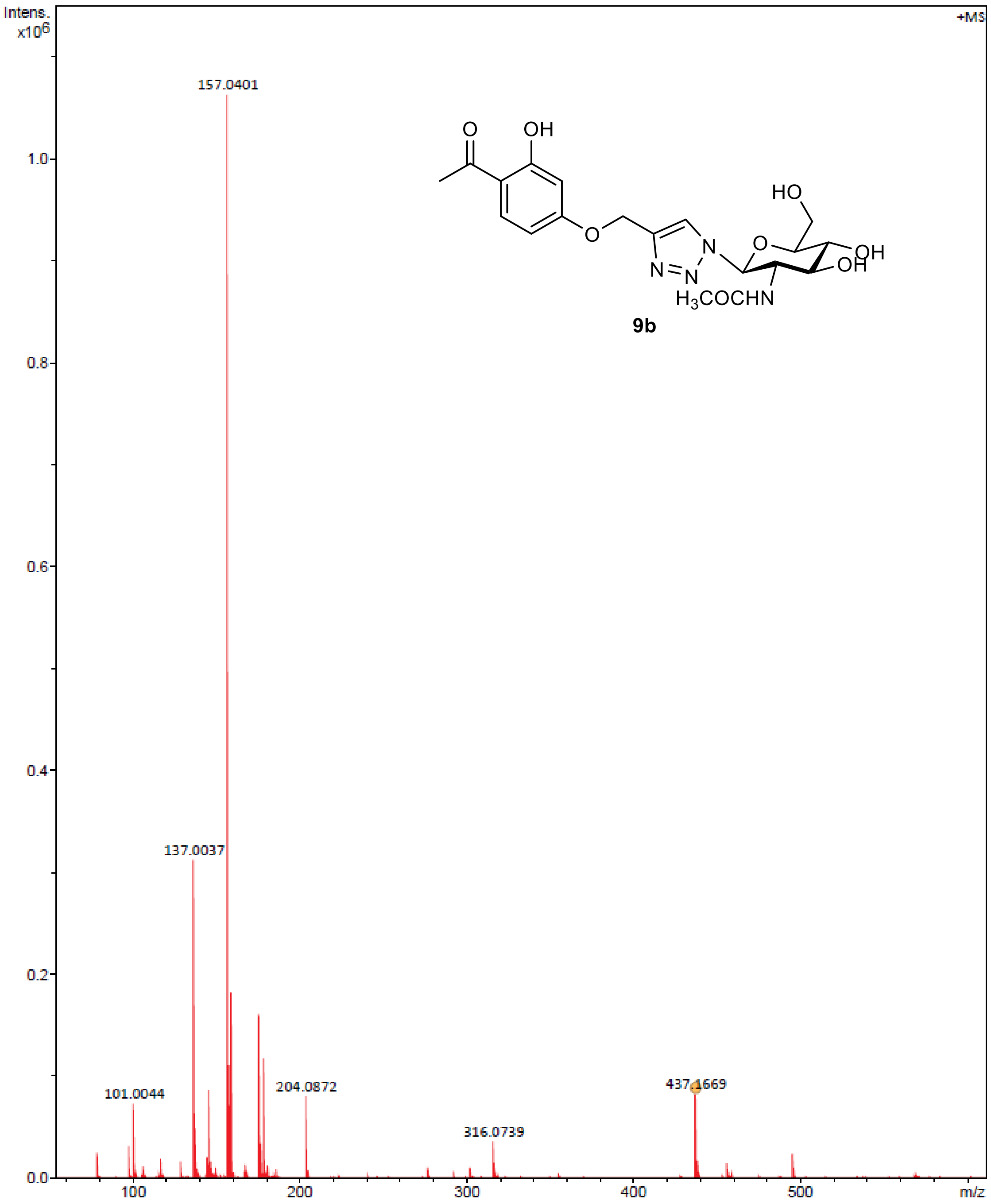

Mass Spectrum Molecular Formula Report

Meas. m/z	#	Ion Formula	m/z	err [ppm]	mSigma	# mSigma	Score	rdb	e⁻ Conf	N-Rule
437.1669	1	C19H25N4O8	437.1667	-0.5	24.8	1	100.00	9.5	even	ok

Figure S28 - HRMS spectrum of compound **9b**.
